# Supplementary material for: High-throughput preparation of radioprotective polymers via Hantzsch’s reaction for in vivo X-ray damage determination
Source: Nat Commun. 2020 Dec 4;11:6214. doi: 10.1038/s41467-020-20027-0 (PMC7718248; doi:10.1038/s41467-020-20027-0)
Supplement: Supplementary file 1 — Supplementary Information [file 41467_2020_20027_MOESM1_ESM.pdf]

Supplementary Information

High-Throughput Preparation of Radioprotective Polymers via Hantzsch's Reaction for In Vivo X-ray Damage Determination

Guoqiang Liu[1], Yuan Zeng[1], Tong Lv[2], Tengfei Mao[1,3], Yen Wei[1], Shunji Jia[2], Yanzi Gou[3], Lei Tao*[1]

[1] The Key Laboratory of Bioorganic Phosphorus Chemistry & Chemical Biology (Ministry of Education), Department of Chemistry, Tsinghua University, Beijing 100084, P. R. China. Email: leitao@mail.tsinghua.edu.cn.

[2] State Key Laboratory of Membrane Biology, Tsinghua-Peking Center for Life Sciences, School of Life Sciences, Tsinghua University, 100084 Beijing, China.

[3] Science and Technology on Advanced Ceramic Fibers and Composites Laboratory, National University of Defense Technology, Changsha, 410073, P. R. China.

1. Materials

All chemicals and solvents were purchased from commercial sources and used without further purification. 2-(Acetoacetoxy)ethyl methacrylate (AEMA, Aladdin, 95%), benzaldehyde (Aladdin, > 99.5%), 4-hydroxy benzaldehyde (TCI, 98%), methyl 4-formylbenzoate (Huawei, 98%), 4-dimethylaminobenzaldehyde (J&K, 99%), cinnamic aldehyde (MREDA, 99%), vanillin (Heowns, 98%), *N*-hexanal (Innochem, 97%), ferrocenecarboxaldehyde (Innochem, > 98%), *p*-nitrobenzaldehyde (Innochem, 98%), 5,5-dimethyl-1,3-cyclohexanedione (MAKLIN, 99%), 1,3-cyclohexanedione

(Shaoyuan, 97%), 5-methyl-1,3-cyclohexanedione (NineDing, 98%), 5-phenyl-1,3-cyclohexanedione (Energy, 97%), 4,4-dimethyl-1,3-cyclohexanedione (TCI, 99%), ammonium acetate (MAKLIN, 98%), glycine (Ouhe, 98%), 2,2'-azobisisoheptonitrile (ABVN, Energy, 98%), poly(ethylene glycol) methyl ether methacrylate (PEGMA, M_n~950 g mol^{-1}, Sigma, 99%), ferrous sulfate (Taida, > 98%), salicylic acid (Innochem, 99%), hydrogen peroxide (Lange, 30% in H_2O), nitrotetrazolium blue chloride (NBT, Dibai, 98%), xanthine (Solarbio, 98%), galvinoxyl (Energy, 98%), amifostine, (Twbio, 98%), WR-1065 (MCR, 99%).

Roswell Park Memorial Institute-1640 (RPMI-1640) culture medium (Corning-Cellgro), phosphate buffered saline (PBS, pH~7.2, 0.01 M, Solarbio), fetal bovine serum (FBS, Gibco), penicillin-streptomycin solution (Gibco), trypsin-EDTA (Gibco, 0.25%), fluorescein diacetate (FDA, Sigma, 99%), propidium iodide (PI, Sigma, 94%), Cell Counting kit-8 (CCK-8, Beyotime, 10%), LMAgarose (1%, Trevigen), lysis solution (Trevigen), Acridine Orange (J&K, 98%), Phospho-Histone H2AX (Ser139) Rabbit Monoclonal Antibody (Bytotime), Alexa Fluor 555-Labeled Donkey Anti-Rabbit IgG (Bytotime), 2,7-dichlorodihydro-fluorescein diacetate (DCFH-DA, 10 mM, Bytotime), crystal violet (Aladdin, 97%) and paraformaldehyde (4%, Bytotime) were used as purchased.

2. Instruments

Gel permeation chromatography (GPC) analyses of polymers were performed using *N*, *N*-dimethyl formamide (DMF) containing 50 mM LiBr as the eluent. The GPC system is a Shimadzu LC-20AD pump system consisting of an auto injector, a MZ-Gel

SDplus 10.0 μm guard column (50 × 8.0 mm, 10^2 Å) followed by two PLgel 5 μm MIXED-D columns (300 × 7.5 mm), and a Shimadzu RID-10A refractive index detector. The system was calibrated with narrow molecular weight distribution polystyrene standards ranging from 200 to 10^6 g mol^{-1}. ^1H NMR spectra were obtained using a JEOL JNM-ECA400 (400 MHz) spectrometer for all samples. Delta 5.3.1 was used to collect NMR data, MestReNova 12.0 was used to analysis NMR data. The ESI-MS data were collected using a Micro TOF-QII Bruker. The FT-IR spectra were recorded in a transmission mode on a Perkin-Elmer Spectrum 100 spectrometer (Waltham, MA, USA).

A Fluorescence microscope (Leica Germany) was used to observe the live and dead cells under 450-490 nm and 515-560 nm band-pass excitation filters (I3 and N2.1), respectively (100 W mercury lamp). Comet Assay ESII Electrophoresis System (Trevigen) was used for single cell gel electrophoresis, the fluorescent images of comet assay, embryo and larvae were collected by a Laser Scanning Confocal Microscopy (LSCM, Zeiss LSM 780), the LSCM files were collected and exported from ZEN black 2.3. Cell viability was measured via a CCK-8 assay by a VICTOR™ X3 PerkinElmer 2030 Multilabel Plate Reader. The cell flow cytometry was carried out by a BD FACS Calibur flow cytometer (BD ArialIII), the results of flow cytometer were analyzed by FlowJo (Version 10, Tree Star, Oregon, USA). The bright field images of larvae were collected by a stereomicroscope (ZSA0850T).

3. Methods

3.1 2-(Methacryloyloxy)ethyl 2,7,7-trimethyl-5-oxo-4-phenyl-1,4,5,6,7,8-

hexahydroquinoline-3-carboxylate (M(1)(1))

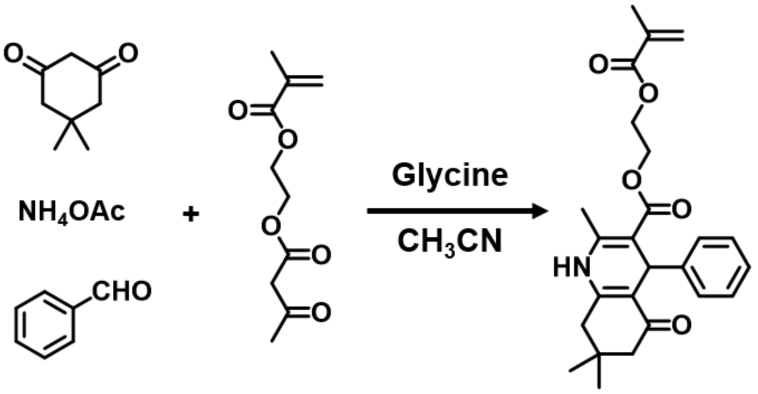

The monomers M(X)(Y) were prepared via the HTP Hantzsch reaction by different combinations of aldehydes (A(X)) and 1,3-cyclohexanedione derivatives (B(Y)). As a typical example, to prepare M(1)(1), benzaldehyde (A(1), 530 mg, 5.0 mmol), AEMA (1.07 g, 5.0 mmol), dimedone (B(1), 700 mg, 5.0 mmol), and ammonium acetate (578 mg, 7.5 mmol) were put in a 15 mL centrifuge tube followed by addition of glycine (38 mg, 0.5 mmol) and acetonitrile (5.0 mL). The mixture was kept in a isothermal shaker (70°C) for 6 h. The crude was washed by water and petroleumether/ethyl acetate (10/1) to get M(1)(1) as a yellow powder (1.94 g, yield: 92 %).

All other monomers were parallelly prepared through the same procedure.

^{1}H-NMR (400 MHz, DMSO-d$_6$, δ/ppm): 9.13 (s, 1H, CNHC), 7.02-7.14 (m, 5H, ph), 5.99 (s, 1H, CH_2=C), 5.69 (s, 1H, CH_2=C), 4.84 (s, 1H, CCHC), 4.17-4.29 (m, 4H, $COOCH_2CH_2$), 2.22-2.41 (m, 2H, CH_2C=O), 2.24 (s, 3H, NHCC$\underline{H}_3$), 1.90-2.16 (m, 2H, C$\underline{H}_2$CNH), 1.82 (s, 3H, C$\underline{H}_3$C=CH_2), 1.00 (s, 3H, C$\underline{H}_3$CCH_3), 0.83 (s, 3H, CH_3CC$\underline{H}_3$). ^{13}C-NMR (100 MHz, DMSO-d$_6$, δ/ppm): 194.80, 167.18, 166.91, 149.95, 148.11, 146.31, 136.15, 128.25, 128.25, 127.90, 127.90, 126.59, 126.23, 110.66, 103.62, 63.22,

61.60, 50.77, 36.27, 32.68, 32.68, 29.67, 26.96, 18.92, 18.47.

IR (ν/cm^{-1}): 3281, 3128, 2212, 2168, 2157, 1965, 1663, 1413, 1328, 1231, 1121, 1092, 924, 905, 860, 793, 669.

ESI-MS: observed (expected): 446.1932 (446.1938) [M + Na^+].

3.2 2-(Methacryloyloxy)ethyl 4-(2,6-dimethylhept-5-en-1-yl)-2,7,7-trimethyl-5-oxo-1,4,5,6,7,8-hexahydroquinoline-3-carboxylate (M(2)(1))

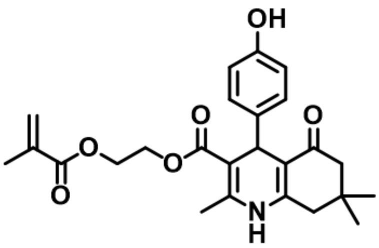

^{1}H-NMR (400 MHz, DMSO-d_6, δ/ppm): 9.04 (s, 1H, CNHC), 6.50-6.90 (m, 4H, ph), 6.01 (s, 1H, CH_2=C), 5.69 (s, 1H, CH_2=C), 4.73 (s, 1H, CCHC), 4.17-4.29 (m, 4H, $COOCH_2CH_2$), 2.22-2.42 (m, 2H, CH_2C=O), 2.23 (s, 3H, NHCC$\underline{H}_3$), 1.91-2.15 (m, 2H, C$\underline{H}_2$CNH), 1.82 (s, 3H, C$\underline{H}_3$C=CH_2), 1.00 (s, 3H, C$\underline{H}_3$CCH_3), 0.83(s, 3H, CH_3CC$\underline{H}_3$).

^{13}C-NMR (100 MHz, DMSO-d_6, δ/ppm): 194.82, 167.34, 166.94, 155.82, 149.55, 145.68, 138.86, 136.16, 128.77, 128.77, 126.59, 126.59, 114.96, 111.01, 104.15, 63.26, 61.55, 50.84, 35.21, 32.68, 32.68, 29.71, 27.00, 18.90, 18.48.

IR (ν/cm^{-1}): 3055, 2183, 2180, 2168, 2126, 2037, 1957, 1710, 1663, 1631, 1523, 1498, 1419, 1331, 1287, 1240, 1194, 1062, 961, 906, 891, 872, 822, 812, 791, 758.

ESI-MS: observed (expected): 462.1886 (462.1887) [M + Na^+].

3.3 2-(Methacryloyloxy)ethyl 4-(4-(methoxycarbonyl)phenyl)-2,7,7-trimethyl-5-oxo-1,4,5,6,7,8-hexahydroquinoline-3-carboxylate (M(3)(1))

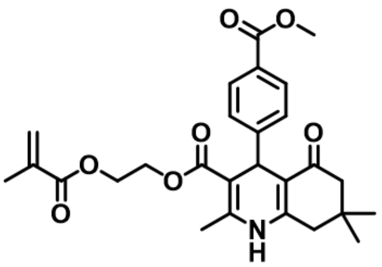

^{1}H-NMR (400 MHz, DMSO-d_6, δ/ppm): 9.21 (s, 1H, CNHC), 7.26-7.74 (m, 4H, ph), 5.93 (s, 1H, CH_2=C), 5.64 (s, 1H, CH_2=C), 4.91 (s, 1H, CCHC), 4.14-4.25 (m, 4H, $COOCH_2CH_2$), 3.80 (s, 3H, OCH_3), 2.15-2.45 (m, 2H, CH_2C=O), 2.30 (s, 3H, $NHCC\underline{H}_3$), 1.82-1.98 (m, 2H, $C\underline{H}_2CNH$), 1.81 (s, 3H, $C\underline{H}_3C$=CH_2), 1.00 (s, 3H, $C\underline{H}_3CCH_3$), 0.80 (s, 3H, $CH_3CC\underline{H}_3$).

^{13}C-NMR (100 MHz, DMSO-d_6, δ/ppm): 194.78, 166.89, 166.82, 166.68, 153.34, 150.21, 147.04, 136.08, 129.36, 129.36, 128.29, 128.29, 127.68, 126.48, 110.13, 102.82, 63.27, 61.61, 52.43, 50.65, 36.80, 32.67, 32.67, 29.64, 26.81, 18.93, 18.39.

IR (ν/cm^{-1}): 2211, 2180, 2167, 2154, 2043, 2032, 2008, 1979, 1958, 1530, 1422, 983, 939, 909, 831, 796.

ESI-MS: observed (expected): 504.1993 (504.1992) [M + Na^+].

3.4 2-(Methacryloyloxy)ethyl 4-(4-(dimethylamino)phenyl)-2,7,7-trimethyl-5-oxo-1,4,5,6,7,8-hexahydroquinoline-3-carboxylate (M(4)(1))

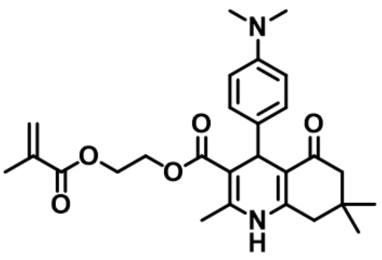

^{1}H-NMR (400 MHz, DMSO-d_6, δ/ppm): 9.02 (s, 1H, CNHC), 6.50-6.94 (m, 4H, ph), 6.01 (s, 1H, CH_2=C), 5.69 (s, 1H, CH_2=C), 4.71 (s, 1H, CCHC), 4.15-4.30 (m, 4H,

$COOCH_2CH_2$), 2.75 (s, 6H, CH_3NCH_3), 2.25-2.44 (m, 2H, $CH_2C{=}O$), 2.25 (s, 3H, $NHCC\underline{H}_3$), 1.93-2.17 (m, 2H, $C\underline{H}_2CNH$), 1.86 (s, 3H, $C\underline{H}_3C{=}CH_2$), 1.00 (s, 3H, $C\underline{H}_3CCH_3$), 0.87 (s, 3H, $CH_3CC\underline{H}_3$).

^{13}C-NMR (100 MHz, DMSO-d_6, δ/ppm): 194.82, 167.41, 166.95, 149.44, 149.27, 149.18, 145.42, 136.55, 136.17, 128.41, 128.41, 126.60, 112.53, 111.04, 104.32, 63.30, 61.53, 61.53, 50.86, 35.02, 32.68, 32.68, 29.73, 27.11, 18.90, 18.50, 18.50.

IR (ν/cm^{-1}): 3675, 3377, 2211, 2192, 2186, 2171, 2167, 2161, 2157, 2124, 2049, 2031, 2021, 2001, 1995, 1979, 1965, 1943, 1892, 1704, 1663, 1601, 1559, 1461, 1333, 1157, 965, 891.

ESI-MS: observed (expected): 489.2360 (489.2356) [M + Na^+].

3.5 2-(Methacryloyloxy)ethyl (E)-2,7,7-trimethyl-5-oxo-4-(4-styrylphenyl)-1,4,5,6,7,8-hexahydroquinoline-3-carboxylate (M(5)(1))

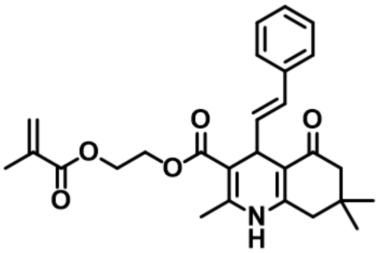

^1H-NMR (400 MHz, DMSO-d_6, δ/ppm): 9.10 (s, 1H, CNHC), 7.04-7.27 (m, 5H, ph), 6.08 (s, 1H, $phC\underline{H}{=}CH$), 6.07 (s, 1H, $phCH{=}C\underline{H}$), 5.99 (s, 1H, $CH_2{=}C$), 5.61 (s, 1H, $CH_2{=}C$), 4.47 (s, 1H, CCHC), 4.22-4.37 (m, 4H, $COOCH_2CH_2$), 2.13-2.41 (m, 2H, $CH_2C{=}O$), 2.28 (s, 3H, $NHCC\underline{H}_3$), 1.82-1.93 (m, 2H, $C\underline{H}_2CNH$), 1.83 (s, 3H, $C\underline{H}_3C{=}CH_2$), 0.93-1.13 (m, 6H, CH_3CCH_3).

^{13}C-NMR (100 MHz, DMSO-d_6, δ/ppm): 197.13, 167.23, 164.74, 146.93, 139.16, 137.36, 135.94, 135.41, 129.02, 126.51, 126.07, 125.03, 124.04, 110.04, 103.57, 62.36,

51.98, 46.55, 40.24, 33.78, 32.40, 30.03, 28.36, 25.31, 24.16, 17.87, 15.22.

IR (ν/cm^{-1}): 3675, 3558, 3287, 3138, 2212, 2196, 2152, 2126, 2037, 2031, 2021, 1992, 1985, 1978, 1965, 1704, 1665, 1549, 1460, 1342, 975, 826, 814.

ESI-MS: observed (expected): 472.2098 (472.2094) [M + Na$^+$].

3.6 2-(Methacryloyloxy)ethyl 4-(4-hydroxy-3-methoxyphenyl)-2,7,7-trimethyl-5-oxo-1,4,5,6,7,8-hexahydroquinoline-3-carboxylate (M(6)(1))

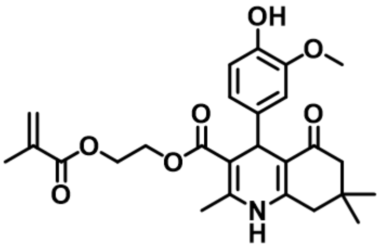

^{1}H-NMR (400 MHz, DMSO-d$_6$, δ/ppm): 9.05 (s, 1H, OH), 8.61 (s, 1H, CNHC), 6.48-6.68 (m, 3H, ph), 5.99 (s, 1H, CH_2=C), 5.67 (s, 1H, CH_2=C), 4.75 (s, 1H, CCHC), 4.20-4.28 (m, 4H, $COOCH_2CH_2$), 3.64 (s, 3H, OCH_3), 2.25-2.45 (m, 2H, CH_2C=O), 2.26 (s, 3H, $NHCC\underline{H}_3$), 1.96-2.19 (m, 2H, $C\underline{H}_2$CNH), 1.86 (s, 3H, $C\underline{H}_3C=CH_2$), 1.00 (s, 3H, $C\underline{H}_3CCH_3$), 0.87 (s, 3H, $CH_3CC\underline{H}_3$).

^{13}C-NMR (100 MHz, DMSO-d$_6$, δ/ppm): 194.93, 167.36, 166.94, 149.67, 147.33, 145.73, 145.12, 139.44, 136.13, 126.55, 120.03, 115.36, 112.35, 110.93, 104.02, 63.21, 61.54, 55.92, 50.83, 35.47, 33.32, 32.67, 29.80, 26.86, 18.90, 18.45.

IR (ν/cm^{-1}): 3675, 3595, 2211, 2206, 2177, 2159, 2151, 2074, 2025, 2018, 2014, 2004, 2000, 1983, 1946, 1538, 1503, 1344, 964, 833.

ESI-MS: observed (expected): 492.1990 (492.1993) [M + Na$^+$].

3.7 2-(Methacryloyloxy)ethyl 2,7,7-trimethyl-5-oxo-4-pentyl-1,4,5,6,7,8-hexahydroquinoline-3-carboxylate (M(7)(1))

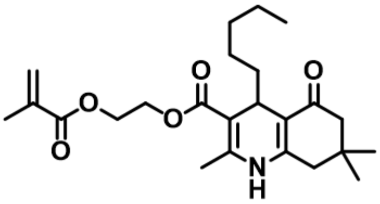

^{1}H-NMR (400 MHz, DMSO-d_6, δ/ppm): 8.88 (s, 1H, CNHC), 6.04 (s, 1H, CH_2=C), 5.69 (s, 1H, CH_2=C), 4.17-4.39 (m, 4H, $COOCH_2CH_2$), 3.79 (s, 1H, CCHC), 2.18-2.40 (m, 2H, CH_2C=O), 2.21 (s, 3H, $NHCC\underline{H}_3$), 2.02-2.18 (m, 2H, $C\underline{H}_2$CNH), 1.89 (s, 3H, $C\underline{H}_3C$=CH_2), 1.04-1.26 (m, 8H, $(C\underline{H}_2)_4CH_3$) 1.02 (s, 3H, $C\underline{H}_3CCH_3$), 0.99 (s, 3H, $CH_3CC\underline{H}_3$), 0.76-0.79 (m, 3H, $CH_2C\underline{H}_3$).

^{13}C-NMR (100 MHz, DMSO-d_6, δ/ppm): 196.14, 167.62, 167.29, 149.90, 145.38, 136.08, 126.15, 111.33, 104.67, 62.81, 61.47, 51.02, 41.08, 36.34, 32.59, 29.84, 29.75, 27.09, 25.01, 22.76, 19.43, 18.36, 14.16, 14.15.

IR (ν/cm^{-1}): 3676, 3585, 3234, 3211, 2188, 2164, 2036, 2013, 1982, 1963, 1949, 1342, 1318, 1186, 1163, 964, 940, 904, 763, 749, 665.

ESI-MS: observed (expected): 440.2405 (440.2407) [M + Na$^+$].

3.8 2-(Methacryloyloxy)ethyl 2,7,7-trimethyl-4-ferrocenyl-5-oxo-1,4,5,6,7,8-hexahydroquinoline-3-carboxylate (M(8)(1))

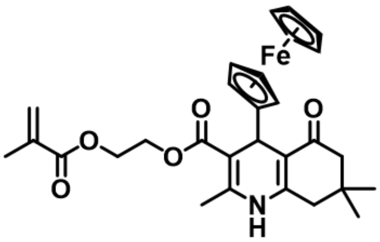

^{1}H-NMR (400 MHz, DMSO-d_6, δ/ppm): 9.17 (s, 1H, $CN\underline{H}C$), 6.08 (s, 1H, CH_2=C), 5.73 (s, 1H, CH_2=C), 4.68 (s, 1H, CCHC), 4.31-4.42 (m, 4H, $COOCH_2CH_2$), 3.69-4.02 (m, 9H, Cp), 2.32-2.42 (m, 2H, CH_2C=O), 2.24 (s, 3H, $NHCC\underline{H}_3$), 2.16-2.19 (m, 2H, $C\underline{H}_2$CNH), 1.91 (s, 3H, $C\underline{H}_3C$=CH_2), 1.03-1.09 (m, 6H, CH_3CCH_3).

^{13}C-NMR (100 MHz, DMSO-d_6, δ/ppm): 195.03, 167.79, 167.00, 150.76, 145.87, 136.20, 129.18, 126.71, 113.65, 111.44, 109.64, 103.81, 96.35, 73.02, 68.85, 66.88, 66.30, 66.30, 66.30, 63.37, 61.87, 60.73, 51.02, 32.46, 30.00, 26.99, 23.27, 18.81, 18.53.

IR (ν/cm^{-1}): 3675, 3413, 3299, 2212, 2187, 2165, 2119, 2009, 1985, 1710, 1658, 1639, 1550, 1430, 1330, 1286, 1186, 1124, 963, 928, 846, 811, 753, 723.

ESI-MS: observed (expected): 554.1593 (554.1601) [M + Na$^+$].

3.9 2-(Methacryloyloxy)ethyl 2,7,7-trimethyl-4-(4-nitrophenyl)-5-oxo-1,4,5,6,7,8-hexahydroquinoline-3-carboxylate (M(9)(1))

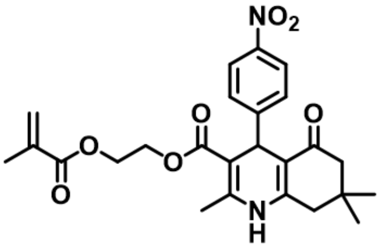

^{1}H-NMR (400 MHz, DMSO-d_6, δ/ppm): 9.30 (s, 1H, CNHC), 7.39-8.05 (m, 4H, ph), 5.89 (s, 1H, CH_2=C), 5.62 (s, 1H, CH_2=C), 4.96 (s, 1H, CCHC), 4.12-4.28 (m, 4H, $COOCH_2CH_2$), 2.28-2.48 (m, 2H, CH_2C=O), 2.33 (s, 3H, NHCC$\underline{H}_3$), 1.87-2.19 (m, 2H, C$\underline{H}_2$CNH), 1.79 (s, 3H, C$\underline{H}_3$C=CH_2), 1.00 (s, 3H, C$\underline{H}_3$CCH_3), 0.81 (s, 3H, CH_3CC$\underline{H}_3$).

^{13}C-NMR (100 MHz, DMSO-d_6, δ/ppm): 194.81, 166.77, 166.70, 155.47, 150.49, 147.69, 147.55, 146.28, 146.11, 136.07, 129.19, 126.42, 123.66, 109.76, 102.36, 63.13, 61.65, 50.58, 37.07, 32.69, 29.54, 26.93, 18.95, 18.34, 18.33.

IR (ν/cm^{-1}): 3675, 3559, 2187, 2170, 2154, 2021, 1977, 1964, 1664, 1557, 1515, 1426, 1378, 1358, 1217, 1198, 1094, 940, 924, 867, 843, 791.

ESI-MS: observed (expected): 491.1786 (491.1789) [M + Na$^+$].

3.10 2-(Methacryloyloxy)ethyl 2-methyl-5-oxo-4-phenyl-1,4,5,6,7,8-

hexahydroquinoline-3-carboxylate (M(1)(2))

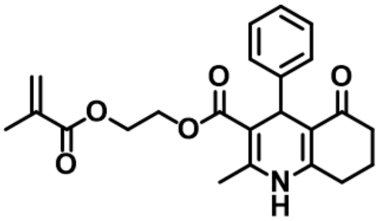

^{1}H-NMR (400 MHz, DMSO-d_6, δ/ppm): 9.20 (s, 1H, CNHC), 7.04-7.26 (m, 5H, ph), 5.99 (s, 1H, CH_2=C), 5.69 (s, 1H, CH_2=C), 4.89 (s, 1H, CCHC), 4.16-4.26 (m, 4H, $COOCH_2CH_2$), 2.44-2.48 (m, 2H, CH_2C=O), 2.27 (s, 3H, NHCC$\underline{H}_3$), 2.17-2.23 (m, 2H, C$\underline{H}_2$CNH), 1.86 (s, 3H, C$\underline{H}_3$C=CH_2), 1.69-1.80 (m, 2H, CH_2C$\underline{H}_2$$CH_2$).

^{13}C-NMR (100 MHz, DMSO-d_6, δ/ppm): 195.18, 167.23, 166.91, 151.85, 148.24, 146.24, 136.15, 128.34, 128.04, 127.85, 126.60, 126.20, 112.98, 111.77, 103.55, 63.23, 61.60, 39.61, 37.25, 36.02, 26.65, 21.33, 18.47.

IR (ν/cm^{-1}): 3129, 2188, 2183, 2158, 2134, 2033, 1988, 1981, 1663, 1541, 1404, 1346, 985, 928, 902, 891, 866, 815, 737, 663.

ESI-MS: observed (expected): 418.1625 (418.1625) [M + Na^+].

3.11 2-(Methacryloyloxy)ethyl 4-(4-hydroxyphenyl)-2-methyl-5-oxo-1,4,5,6,7,8-hexahydroquinoline-3-carboxylate (M(2)(2))

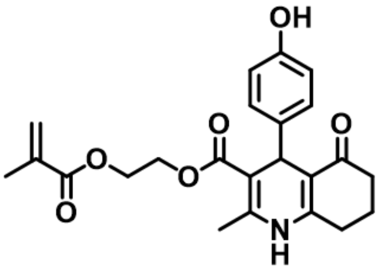

^{1}H-NMR (400 MHz, DMSO-d_6, δ/ppm): 9.12 (s, 1H, CNHC), 6.50-6.91 (m, 4H, ph), 6.01 (s, 1H, CH_2=C), 5.69 (s, 1H, CH_2=C), 4.77 (s, 1H, CCHC), 4.16-4.27 (m, 4H, $COOCH_2CH_2$), 2.44-2.48 (m, 2H, CH_2C=O), 2.26 (s, 3H, NHCC$\underline{H}_3$), 2.15-2.21 (m, 2H,

C$\underline{H}_2$CNH), 1.87 (s, 3H, C$\underline{H}_3$C=CH_2), 1.70-1.81 (m, 2H, CH_2C$\underline{H}_2$$CH_2$).

^{13}C-NMR (100 MHz, DMSO-d_6, δ/ppm): 195.20, 167.39, 166.94, 155.85, 151.47, 145.62, 138.93, 138.57, 136.15, 128.71, 126.59, 115.03, 113.42, 112.13, 104.09, 63.27, 61.55, 39.40, 37.31, 34.95, 21.37, 18.86, 18.48.

IR (ν/cm^{-1}): 3366, 3055, 2972, 2161, 2150, 2036, 2010, 1693, 1660, 1525, 1500, 1418, 1343, 1268, 1202, 1094, 988, 976, 815, 721.

ESI-MS: observed (expected): 434.1570 (434.1574) [M + Na^+].

3.12 2-(Methacryloyloxy)ethyl 4-(4-(methoxycarbonyl)phenyl)-2-methyl-5-oxo-1,4,5,6,7,8-hexahydroquinoline-3-carboxylate (M(3)(2))

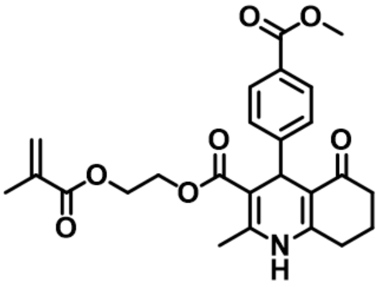

^{1}H-NMR (400 MHz, DMSO-d_6, δ/ppm): 9.29 (s, 1H, CNHC), 7.26-7.76 (m, 4H, ph), 5.93 (s, 1H, CH_2=C), 5.64 (s, 1H, CH_2=C), 4.95 (s, 1H, CCHC), 4.14-4.32 (m, 4H, $COOCH_2CH_2$), 3.80 (s, 3H, OCH_3), 2.46-2.49 (m, 2H, CH_2C=O), 2.30 (s, 3H, NHCC$\underline{H}_3$), 2.14-2.24 (m, 2H, C$\underline{H}_2$CNH), 1.81 (s, 3H, C$\underline{H}_3$C=CH_2), 1.70-1.77 (m, 2H, CH_2C$\underline{H}_2$$CH_2$).

^{13}C-NMR (100 MHz, DMSO-d_6, δ/ppm): 195.16, 166.94, 166.82, 166.69, 153.54, 152.23, 152.14, 146.94, 136.08, 129.43, 128.26, 127.68, 126.48, 112.33, 111.25, 102.76, 63.18, 61.60, 52.43, 37.16, 36.59, 26.86, 26.64, 18.89, 18.39.

IR (ν/cm^{-1}): 2957, 2210, 2188, 2169, 2141, 2029, 2022, 1999, 1948, 1538, 1459, 1400, 1348, 1204, 1025, 988, 923, 830, 793, 738, 674.

ESI-MS: observed (expected): 476.1680 (476.1680) [M + Na^+].

3.13 2-(Methacryloyloxy)ethyl 4-(4-(dimethylamino)phenyl)-2-methyl-5-oxo-1,4,5,6,7,8-hexahydroquinoline-3-carboxylate (M(4)(2))

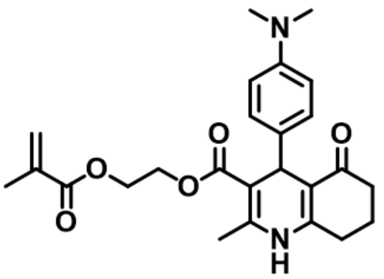

^{1}H-NMR (400 MHz, DMSO-d_6, δ/ppm): 9.09 (s, 1H, CNHC), 6.51-6.94 (m, 4H, ph), 6.01 (s, 1H, CH_2=C), 5.69 (s, 1H, CH_2=C), 4.76 (s, 1H, CCHC), 4.16-4.29 (m, 4H, $COOCH_2CH_2$), 2.78 (s, 6H, CH_3NCH_3), 2.43-2.48 (m, 2H, CH_2C=O), 2.25 (s, 3H, $NHCC\underline{H}_3$), 2.14-2.21 (m, 2H, $C\underline{H}_2$CNH), 1.86 (s, 3H, $C\underline{H}_3C=CH_2$), 1.69-1.79 (m, 2H, $CH_2C\underline{H}_2CH_2$).

^{13}C-NMR (100 MHz, DMSO-d_6, δ/ppm): 195.18, 167.45, 166.94, 151.27, 149.23, 145.42, 136.67, 136.16, 128.33, 126.78, 126.60, 113.53, 112.70, 112.24, 104.19, 63.29, 61.53, 37.33, 34.77, 26.86, 26.64, 21.40, 18.85, 18.50, 18.50.

IR (ν/cm^{-1}): 2675, 2220, 2182, 2148, 2038, 2018, 2003, 1716, 1703, 1544, 1436, 1304, 1292, 1163, 1079, 972, 858, 781, 769, 722, 687.

ESI-MS: observed (expected): 461.2046 (461.2047) [M + Na^+].

3.14 2-(Methacryloyloxy)ethyl (E)-2-methyl-5-oxo-4-(4-styrylphenyl)-1,4,5,6,7,8-hexahydroquinoline-3-carboxylate (M(5)(2))

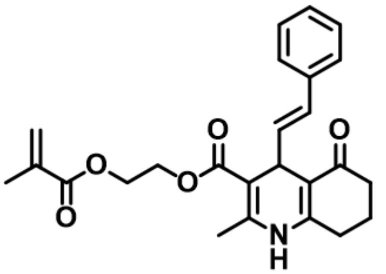

^{1}H-NMR (400 MHz, DMSO-d_6, δ/ppm): 9.17 (s, 1H, CNHC), 7.16-7.26 (m, 5H, ph), 6.09 (m, 1H, phC$\underline{H}$=CH), 6.03 (m, 1H, phCH=C$\underline{H}$), 5.99 (s, 1H, CH_2=C), 5.62 (s, 1H, CH_2=C), 4.50 (s, 1H, CCHC), 4.23-4.36 (m, 4H, $COOCH_2CH_2$), 2.44-2.49 (m, 2H, CH_2C=O), 2.26 (s, 3H, NHCC$\underline{H}_3$), 2.22-2.25 (m, 2H, C$\underline{H}_2$CNH), 1.82 (s, 3H, C$\underline{H}_3$C=CH_2), 1.76-1.79 (m, 2H, CH_2C$\underline{H}_2$$CH_2$).

^{13}C-NMR (100 MHz, DMSO-d_6, δ/ppm): 195.27, 168.71, 167.12, 166.93, 166.89, 152.65, 147.09, 137.62, 136.15, 132.90, 128.97, 126.47, 109.25, 101.15, 99.41, 63.26, 61.69, 57.56, 37.28, 34.09, 32.93, 26.70, 21.46, 18.90, 18.42.

IR (ν/cm^{-1}): 3663, 3126, 2213, 2174, 2161, 2041, 1984, 1698, 1607, 1459, 1417, 1289, 1221, 1128, 1099, 973, 891, 828, 797, 753, 698.

ESI-MS: observed (expected): 444.1781 (444.1781) [M + Na^+].

3.15 2-(Methacryloyloxy)ethyl 4-(4-hydroxy-3-methoxyphenyl)-2-methyl-5-oxo-1,4,5,6,7,8-hexahydroquinoline-3-carboxylate (M(6)(2))

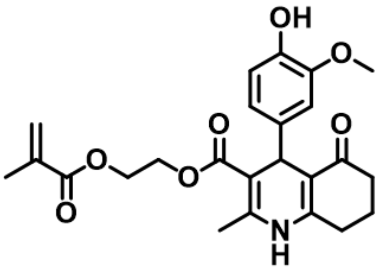

^{1}H-NMR (400 MHz, DMSO-d_6, δ/ppm): 9.77 (s, 1H, OH), 9.12 (s, 1H, CNHC), 6.42-6.70 (m, 3H, ph), 5.99 (s, 1H, CH_2=C), 5.67 (s, 1H, CH_2=C), 4.76 (s, 1H, CCHC), 4.16-

4.29 (m, 4H, $COOCH_2CH_2$), 3.66 (s, 3H, OCH_3), 2.45-2.48 (m, 2H, $CH_2C{=}O$), 2.26 (s, 3H, $NHCC\underline{H}_3$), 2.16-2.23 (m, 2H, $C\underline{H}_2CNH$), 1.86 (s, 3H, $C\underline{H}_3C{=}CH_2$), 1.71-1.82 (m, 2H, $CH_2C\underline{H}_2CH_2$).

^{13}C-NMR (100 MHz, DMSO-d_6, δ/ppm): 195.30, 167.42, 166.94, 151.50, 147.33, 145.67, 145.14, 139.49, 136.12, 119.88, 115.50, 113.23, 112.32, 112.02, 103.91, 63.20, 61.53, 55.89, 37.33, 35.22, 26.66, 21.40, 18.86, 18.44.

IR (ν/cm^{-1}): 3581, 3125, 2901, 2193, 2163, 2037, 2027, 2009, 1966, 1659, 1511, 1474, 1420, 1344, 1226, 1174, 1095, 985, 946, 892, 744, 668.

ESI-MS: observed (expected): 464.1679 (464.1680) [M + Na^+].

3.16 2-(Methacryloyloxy)ethyl 4-hexyl-2-methyl-5-oxo-1,4,5,6,7,8-hexahydroquinoline-3-carboxylate (M(7)(2))

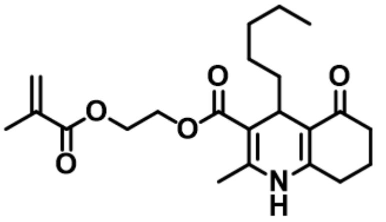

^{1}H-NMR (400 MHz, DMSO-d_6, δ/ppm): 8.97 (s, 1H, CNHC), 6.04 (s, 1H, $CH_2{=}C$), 5.69 (s, 1H, $CH_2{=}C$), 4.30-4.39 (m, 4H, $COOCH_2CH_2$), 3.81 (s, 1H, CCHC), 2.36-2.44 (m, 2H, $CH_2C{=}O$), 2.19 (s, 3H, $NHCC\underline{H}_3$), 1.88 (s, 3H, $C\underline{H}_3C{=}CH_2$), 1.73-1.83 (m, 2H, $C\underline{H}_2CNH$), 1.71-1.82 (m, 2H, $C\underline{H}_2CH_2C{=}O$), 1.00-1.22 (m, 8H, $(C\underline{H}_2)_4CH_3$), 0.78 (t, 3H, J = 6.9 Hz, 6.9 Hz, $CH_2C\underline{H}_3$).

^{13}C-NMR (100 MHz, DMSO-d_6, δ/ppm): 195.55, 167.51, 166.91, 152.69, 146.80, 136.22, 126.50, 110.90, 102.88, 63.29, 61.43, 37.38, 36.58, 32.06, 29.57, 26.69, 24.25, 22.61, 21.52, 18.79, 18.44, 14.42.

IR (ν/cm^{-1}): 3663, 3280, 3128, 3042, 2927, 2233, 2189, 2172, 2154, 2038, 2012, 1607, 1484, 1348, 1306, 1157, 1107, 1056, 965, 864, 814, 779, 710.

ESI-MS: observed (expected): 412.2096 (412.2094) [M + Na$^+$].

3.17 2-(Methacryloyloxy)ethyl -2-methyl-4-ferrocenyl-5-oxo-1,4,5,6,7,8-hexahydroquinoline-3-carboxylate (M(8)(2))

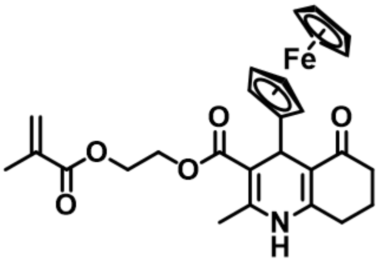

^{1}H-NMR (400 MHz, DMSO-d$_6$, δ/ppm): 9.25 (s, 1H, CNHC), 6.08 (s, 1H, CH_2=C), 5.73 (s, 1H, CH_2=C), 4.70 (s, 1H, CCHC), 4.33-4.42 (m, 4H, $COOCH_2CH_2$), 3.72-4.00 (m, 9H, Cp), 2.37-2.48 (m, 2H, CH_2C=O), 2.19-2.27 (m, 2H, C$\underline{H}_2$CNH), 2.18 (s, 3H, NHCC$\underline{H}_3$), 1.86 (s, 3H, C$\underline{H}_3$C=CH_2), 1.78-1.82 (m, 2H, CH_2C$\underline{H}_2$$CH_2$).

^{13}C-NMR (100 MHz, DMSO-d$_6$, δ/ppm): 195.40, 167.68, 167.00, 152.31, 145.64, 145.64， 136.19, 136.18, 126.70, 126.70, 110.66, 104.23, 96.74, 87.22, 68.86, 66.89, 66.82, 66.09, 63.40, 61.81, 37.48, 28.16, 26.81, 21.39, 18.74, 18.53.

IR (ν/cm^{-1}): 3675, 3290, 2934, 2209, 2176, 2160, 2041, 2021, 1990, 1667, 1479, 1451, 1344, 1217, 1168, 1097, 1061, 950, 838, 805, 772, 738.

ESI-MS: observed (expected): 526.1296 (526.1288) [M + Na$^+$].

3.18 2-(Methacryloyloxy)ethyl 2-methyl-4-(4-nitrophenyl)-5-oxo-1,4,5,6,7,8-hexahydroquinoline-3-carboxylate (M(9)(2))

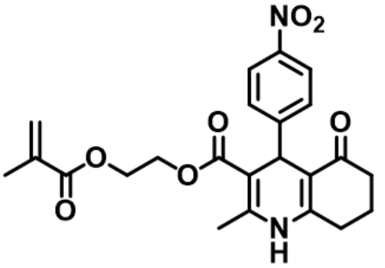

^{1}H-NMR (400 MHz, DMSO-d$_6$, δ/ppm): 9.37 (s, 1H, CNHC), 7.40-8.04 (m, 4H, ph), 5.89 (s, 1H, CH_2=C), 5.62 (s, 1H, CH_2=C), 5.00 (s, 1H, CCHC), 4.02-4.32 (m, 4H, $COOCH_2CH_2$), 2.23-2.46 (m, 2H, CH_2C=O), 2.29 (s, 3H, NHCC$\underline{H}_3$), 2.10-2.20 (m, 2H, C$\underline{H}_2$CNH), 1.75 (s, 3H, C$\underline{H}_3$C=CH_2), 1.64-1.72 (m, 2H, CH_2C$\underline{H}_2$$CH_2$).

^{13}C-NMR (100 MHz, DMSO-d$_6$, δ/ppm): 195.32, 195.17, 166.73, 155.68, 152.54, 152.40, 147.46, 146.12, 136.07, 129.16, 126.42, 123.72, 111.94, 110.91, 102.27, 63.14, 61.65, 37.08, 36.86, 26.63, 21.21, 18.92, 18.34.

IR (ν/cm^{-1}): 3676, 2970, 2177, 2151, 2033, 2005, 1990, 1971, 1704, 1663, 1549, 1503, 1484, 1379, 1343, 1267, 1201, 1127, 1107, 1076, 858, 842, 792.

ESI-MS: observed (expected): 463.1476 (463.1476) [M + Na$^+$].

3.19 2-(Methacryloyloxy)ethyl 2,7-dimethyl-5-oxo-4-phenyl-1,4,5,6,7,8-hexahydroquinoline-3-carboxylate (M(1)(3))

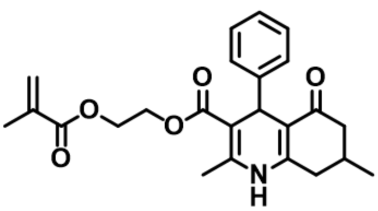

^{1}H-NMR (400 MHz, DMSO-d$_6$, δ/ppm): 9.19 (s, 1H, CNHC), 7.01-7.20 (m, 5H, ph), 5.99 (s, 1H, CH_2=C), 5.68 (s, 1H, CH_2=C), 4.88 (s, 1H, CCHC), 4.15-4.31 (m, 4H, $COOCH_2CH_2$), 2.23 (s, 3H, NHCC$\underline{H}_3$), 2.12-2.18 (m, 2H, CH_2C=O), 1.90-1.98 (m, 2H, C$\underline{H}_2$CNH), 1.82 (s, 3H, C$\underline{H}_3$C=CH_2), 0.95-0.97 (d, 3H, J = 5.2 Hz, CHC$\underline{H}_3$), 0.87-0.90

(m, 1H, C<u>H</u>CH_3).

^{13}C-NMR (100 MHz, DMSO-d_6, δ/ppm): 195.23, 194.81, 167.23, 166.91, 151.45, 150.74, 148.22, 146.24, 136.14, 129.81, 127.80, 126.60, 126.21, 111.44, 103.64, 63.23, 61.61, 45.48, 36.00, 28.63, 21.16, 20.80, 18.91, 18.47.

IR (ν/cm^{-1}): 3286, 3078, 2185, 2168, 2137, 2029, 1986, 1709, 1606, 1418, 1346, 1295, 1275, 1194, 1115, 1017, 947, 885, 826, 795, 699.

ESI-MS: observed (expected): 432.1782 (432.1781) [M + Na^+].

3.20 2-(Methacryloyloxy)ethyl 4-(4-hydroxyphenyl)-2,7-dimethyl-5-oxo-1,4,5,6,7,8-hexahydroquinoline-3-carboxylate (M(2)(3))

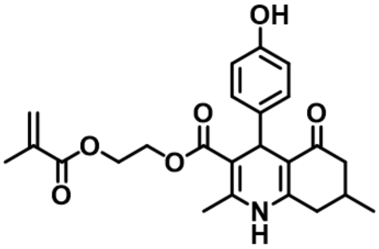

^{1}H-NMR (400 MHz, DMSO-d_6, δ/ppm): 9.03 (s, 1H, CNHC), 6.47-6.93 (m, 4H, ph), 6.01 (s, 1H, CH_2=C), 5.69 (s, 1H, CH_2=C), 4.76 (s, 1H, CCHC), 4.15-4.31 (m, 4H, $COOCH_2CH_2$), 2.21 (s, 3H, NHCC<u>H</u>3), 2.08-2.17 (m, 2H, CH_2C=O), 1.87-1.98 (m, 2H, C<u>H</u>2CNH), 1.83 (s, 3H, C<u>H</u>3C=CH_2), 0.93-0.97 (d, 3H, *J* = 5.7 Hz, CHC<u>H</u>3), 0.87-0.92 (m, 1H, C<u>H</u>CH_3).

^{13}C-NMR (100 MHz, DMSO-d_6, δ/ppm): 195.24, 167.39, 166.94, 155.85, 151.07, 150.34, 145.62, 138.91, 136.15, 128.68, 126.60, 115.03, 114.91, 111.78, 104.19, 63.27, 61.55, 45.56, 35.24, 28.99, 21.19, 20.84, 18.89, 18.49.

IR (ν/cm^{-1}): 3418, 3282, 2211, 2186, 2127, 2009, 1702, 1655, 1512, 1481, 1346, 1282, 1214, 1128, 1104, 1044, 995, 911, 851, 840, 791, 754.

ESI-MS: observed (expected): 448.1735 (448.1731) [M + Na^+].

3.21 2-(Methacryloyloxy)ethyl 4-(4-(methoxycarbonyl)phenyl)-2,7-dimethyl-5-oxo-1,4,5,6,7,8-hexahydroquinoline-3-carboxylate (M(3)(3))

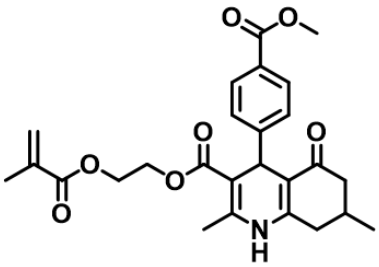

^{1}H-NMR (400 MHz, DMSO-d_6, δ/ppm): 9.29 (s, 1H, CNHC), 7.27-7.76 (m, 4H, ph), 5.93 (s, 1H, CH_2=C), 5.64 (s, 1H, CH_2=C), 4.94 (s, 1H, CCHC), 4.11-4.33 (m, 4H, $COOCH_2CH_2$), 3.80 (s, 3H, OCH_3), 2.30 (s, 3H, $NHCC\underline{H}_3$), 2.15-2.26 (m, 2H, CH_2C=O), 1.89-2.04 (m, 2H, $C\underline{H}_2$CNH), 1.81 (s, 3H, $C\underline{H}_3C=CH_2$), 0.96-1.01 (d, 3H, J = 5.7 Hz, $CHC\underline{H}_3$), 0.88-0.93 (m, 1H, $C\underline{H}CH_3$).

^{13}C-NMR (100 MHz, DMSO-d_6, δ/ppm): 195.21, 194.77, 166.94, 166.69, 153.51, 151.73, 150.99, 146.96, 136.09, 129.44, 128.34, 128.21, 127.69, 126.49, 110.86, 102.84, 63.18, 61.61, 52.44, 36.57, 28.84, 28.57, 21.12, 20.68, 18.92, 18.39.

IR (ν/cm^{-1}): 3288, 2955, 2169, 2151, 2018, 2010, 1976, 1966, 1607, 1482, 1405, 1346, 1216, 1158, 1094, 1025, 948, 865, 798, 737.

ESI-MS: observed (expected): 490.1839 (490.1836) [M + Na^+].

3.22 2-(Methacryloyloxy)ethyl 4-(4-(dimethylamino)phenyl)-2,7-dimethyl-5-oxo-1,4,5,6,7,8-hexahydroquinoline-3-carboxylate (M(4)(3))

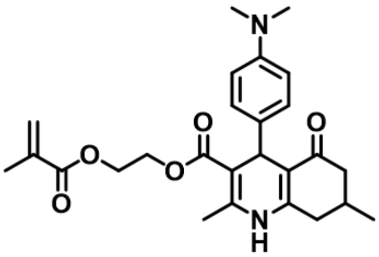

^{1}H-NMR (400 MHz, DMSO-d_6, δ/ppm): 9.12 (s, 1H, CNHC), 6.47-6.94 (m, 4H, ph), 6.01 (s, 1H, CH_2=C), 5.70 (s, 1H, CH_2=C), 4.75 (s, 1H, CCHC), 4.14-4.30 (m, 4H, $COOCH_2CH_2$), 2.78 (s, 6H, CH_3NCH_3), 2.21 (s, 3H, $NHCC\underline{H}_3$), 2.10-2.17 (m, 2H, CH_2C=O), 1.90-1.98 (m, 2H, $C\underline{H}_2$CNH), 1.83 (s, 3H, $C\underline{H}_3C=CH_2$), 0.93-0.97 (d, 3H, J = 5.7 Hz, $CHC\underline{H}_3$), 0.89-0.92 (m, 1H, $C\underline{H}CH_3$).

^{13}C-NMR (100 MHz, DMSO-d_6, δ/ppm): 195.23, 194.82, 167.45, 166.94, 150.88, 150.18, 149.23, 145.42, 136.65, 136.16, 128.30, 126.59, 112.70, 111.90, 104.30, 63.29, 61.53, 45.58, 34.78, 34.67, 29.00, 28.70, 21.20, 20.91, 18.87, 18.50.

IR (ν/cm^{-1}): 3675, 3286, 2938, 2220, 2161, 2108, 2037, 2015, 1973, 1959, 1666, 1546, 1485, 1453, 1336, 1306, 1221, 1163, 1123, 1061, 951, 760, 666.

ESI-MS: observed (expected): 475.2203 (475.2203) [M + Na^+].

3.23 2-(Methacryloyloxy)ethyl (E)-2,7-dimethyl-5-oxo-4-(4-styrylphenyl)-1,4,5,6,7,8-hexahydroquinoline-3-carboxylate (M(5)(3))

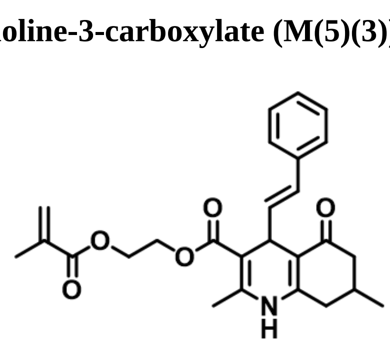

^{1}H-NMR (400 MHz, DMSO-d_6, δ/ppm): 9.16 (s, 1H, CNHC), 7.01-7.33 (m, 5H, ph), 6.05 (m, 1H, $phC\underline{H}=CH$), 6.02 (m, 1H, $phCH=C\underline{H}$), 6.00 (s, 1H, CH_2=C), 5.62 (s, 1H,

$CH_2=C$), 4.47 (s, 1H, CCHC), 4.20-4.40 (m, 4H, $COOCH_2CH_2$), 2.23 (s, 3H, $NHCC\underline{H}_3$), 2.06-2.18 (m, 2H, $CH_2C=O$), 1.93-2.00 (m, 2H, $C\underline{H}_2CNH$), 1.79 (s, 3H, $C\underline{H}_3C=CH_2$), 0.97-1.00 (m, 3H, $CHC\underline{H}_3$), 0.96-0.97 (m, 1H, $C\underline{H}CH_3$).

^{13}C-NMR (100 MHz, DMSO-d_6, δ/ppm): 195.29, 167.11, 166.93, 152.26, 151.61, 147.52, 137.61, 128.97, 127.93, 127.45, 126.42, 109.21, 108.84, 101.30, 100.96, 63.26, 61.61, 45.56, 45.01, 34.15, 32.92, 29.00, 28.75, 21.27, 18.93, 18.44.

IR (ν/cm^{-1}): 3675, 3285, 2916, 2212, 2080, 2159, 2016, 1993, 1716, 1692, 1465, 1379, 1190, 1130, 1085, 1046, 907, 832, 782, 746, 674.

ESI-MS: observed (expected): 458.1938 (458.1938) [M + Na^+].

3.24 2-(Methacryloyloxy)ethyl 4-(4-hydroxy-3-methoxyphenyl)-2,7-dimethyl-5-oxo-1,4,5,6,7,8-hexahydroquinoline-3-carboxylate (M(6)(3))

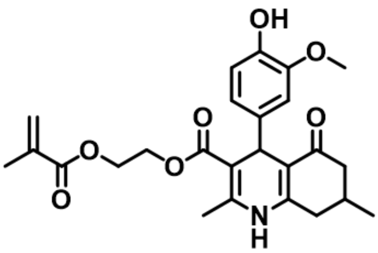

^{1}H-NMR (400 MHz, DMSO-d_6, δ/ppm): 9.12 (s, 1H, OH), 8.61 (s, 1H, CNHC), 6.45-6.68 (m, 3H, ph), 5.99 (s, 1H, $CH_2=C$), 5.67 (s, 1H, $CH_2=C$), 4.79 (s, 1H, CCHC), 4.20-4.28 (m, 4H, $COOCH_2CH_2$), 3.65 (s, 3H, OCH_3), 2.22 (s, 3H, $NHCC\underline{H}_3$), 2.12-2.19 (m, 2H, $CH_2C=O$), 1.90-1.98 (m, 2H, $C\underline{H}_2CNH$), 1.81 (s, 3H, $C\underline{H}_3C=CH_2$), 0.94-0.98 (d, 3H, J = 5.8 Hz, $CHC\underline{H}_3$), 0.88-0.92 (m, 1H, $C\underline{H}CH_3$).

^{13}C-NMR (100 MHz, DMSO-d_6, δ/ppm): 195.32, 167.43, 166.94, 151.11, 150.34, 147.36, 145.67, 145.16, 139.47, 136.14, 126.57, 120.02, 115.51, 112.31, 104.02, 63.20, 61.56, 55.95, 45.58, 35.20, 34.68, 28.68, 21.20, 18.88, 18.45.

IR (ν/cm^{-1}): 3675, 3289, 2932, 2188, 2165, 2148, 2015, 1988, 1699, 1607, 1502, 1439, 1379, 1269, 1192, 1145, 1077, 1056, 914, 838, 779.

ESI-MS: observed (expected): 478.1832 (478.1836) [M + Na$^+$].

3.25 2-(Methacryloyloxy)ethyl 4-hexyl-2,7-dimethyl-5-oxo-1,4,5,6,7,8-hexahydroquinoline-3-carboxylate (M(7)(3))

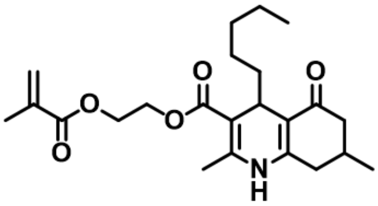

^{1}H-NMR (400 MHz, DMSO-d$_6$, δ/ppm): 8.92 (s, 1H, CNHC), 6.03 (s, 1H, CH_2=C), 5.69 (s, 1H, CH_2=C), 4.16-4.36 (m, 4H, $COOCH_2CH_2$), 3.79 (s, 1H, CCHC), 2.20 (s, 3H, NHCC$\underline{H}_3$), 2.12-2.19 (m, 2H, CH_2C=O), 1.90-1.98 (m, 2H, C$\underline{H}_2$CNH), 1.88 (s, 3H, C$\underline{H}_3$C=CH_2), 1.03-1.20 (m, 8H, (C$\underline{H}_2$)$_4$$CH_3$), 0.94-0.98 (m, 3H, CHC$\underline{H}_3$), 0.88-0.92 (m, 1H, C$\underline{H}$$CH_3$), 0.75-0.81 (m, 3H, CH_2C$\underline{H}_3$).

^{13}C-NMR (100 MHz, DMSO-d$_6$, δ/ppm): 195.55, 195.08, 167.49, 166.89, 152.33, 151.46, 146.98, 136.22, 110.58, 63.27, 61.43, 45.68, 44.99, 32.12, 29.84, 28.71, 24.37, 22.63, 21.22, 20.69, 18.80, 18.43, 14.40.

IR (ν/cm^{-1}): 3675, 3284, 3125, 2943, 2193, 2179, 2150, 2065, 2027, 1991, 1704, 1662, 1604, 1488, 1422, 1349, 1287, 1215, 1144, 1111, 1041, 996, 923, 900, 779, 738, 669.

ESI-MS: observed (expected): 426.2252 (426.2251) [M + Na$^+$].

3.26 2-(Methacryloyloxy)ethyl -2,7-dimethyl-4-ferrocenyl-5-oxo-1,4,5,6,7,8-hexahydroquinoline-3-carboxylate (M(8)(3))

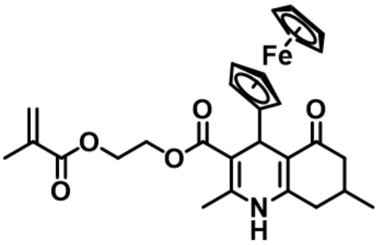

^{1}H-NMR (400 MHz, DMSO-d6, δ/ppm): 9.16 (s, 1H, CNHC), 6.08 (s, 1H, CH_2=C), 5.74 (s, 1H, CH_2=C), 4.67 (s, 1H, CCHC), 4.32-4.42 (m, 4H, $COOCH_2CH_2$), 3.72-4.00 (m, 9H, Cp), 2.33-2.41 (m, 2H, CH_2C=O), 2.24 (s, 3H, NHCC$\underline{H}_3$), 2.08-2.20 (m, 2H, C$\underline{H}_2$CNH), 1.92 (s, 3H, C$\underline{H}_3$C=CH_2), 1.05-1.08 (d, 3H, *J* = 6.0 Hz, CHC$\underline{H}_3$), 1.03-1.05 (m, 1H, C$\underline{H}$$CH_3$).

^{13}C-NMR (100 MHz, DMSO-d6, δ/ppm): 195.02, 167.79, 167.01, 164.69, 154.34, 151.05, 147.80, 145.80, 136.20, 126.70, 125.47, 110.51, 104.39, 103.70, 96.63, 68.87, 66.82, 66.39, 66.27, 63.39, 61.84, 45.08, 34.05, 28.77, 28.18, 20.81, 18.79, 18.53.

IR (ν/cm^{-1}): 3675, 3294, 2934, 2216, 2180, 2165, 2063, 2038, 1701, 1602, 1439, 1389, 1265, 1212, 1126, 1070, 1056, 892, 751.

ESI-MS: observed (expected): 540.1444 (540.1444) [M + Na^+].

3.27 2-(Methacryloyloxy)ethyl 2,7-dimethyl-4-(4-nitrophenyl)-5-oxo-1,4,5,6,7,8-hexahydroquinoline-3-carboxylate (M(9)(3))

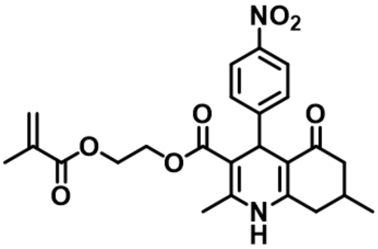

^{1}H-NMR (400 MHz, DMSO-d6, δ/ppm): 9.37 (s, 1H, CNHC), 7.38-8.06 (m, 4H, ph), 5.89 (s, 1H, CH_2=C), 5.62 (s, 1H, CH_2=C), 4.95 (s, 1H, CCHC), 4.04-4.32 (m, 4H, $COOCH_2CH_2$), 2.28 (s, 3H, NHCC$\underline{H}_3$), 2.11-2.25 (m, 2H, CH_2C=O), 1.83-1.98 (m, 2H,

C$\underline{H}_2$CNH), 1.74 (s, 3H, C$\underline{H}_3$C=CH_2), 0.90-0.98 (d, 3H, *J* = 6.0 Hz CHC$\underline{H}_3$), 0.83-0.89 (m, 1H, C$\underline{H}$$CH_3$).

^{13}C-NMR (100 MHz, DMSO-d_6, δ/ppm): 195.23, 194.80, 166.77, 155.65, 151.98, 151.31, 147.44, 146.11, 136.07, 129.24, 126.43, 123.73, 123.62, 110.58, 102.34, 63.14, 61.64, 45.27, 44.84, 37.14, 21.09, 20.73, 18.95, 18.34.

IR (ν/cm^{-1}): 3675, 2972, 2901, 2185, 2168, 2150, 2135, 2025, 2007, 1986, 1966, 1662, 1604, 1515, 1481, 1431, 1359, 1305, 1217, 1159, 1077, 969, 840.

ESI-MS: observed (expected): 477.1630 (477.1632) [M + Na^+].

3.28 2-(Methacryloyloxy)ethyl 2-methyl-5-oxo-4,7-diphenyl-1,4,5,6,7,8-hexahydroquinoline-3-carboxylate (M(1)(4))

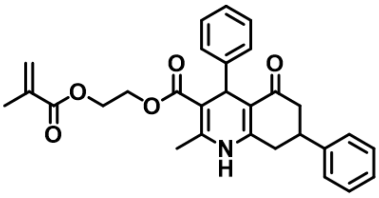

^{1}H-NMR (400 MHz, DMSO-d_6, δ/ppm): 9.18 (s, 1H, CNHC), 7.04-7.37 (m, 10H, ph), 6.00 (s, 1H, CH_2=C), 5.69 (s, 1H, CH_2=C), 4.89 (s, 1H, CCHC), 4.17-4.30 (m, 4H, COO$CH_2$$CH_2$), 2.60-2.86 (m, 2H, C$\underline{H}_2$C=O), 2.31-2.35 (m, 2H, NHCC$\underline{H}_2$), 2.28 (s, 3H, C$\underline{H}_3$CNH), 1.87 (s, 3H, C$\underline{H}_3$C=CH_2), 0.81-1.26 (m, 1H, CH_2C$\underline{H}$$CH_2$).

^{13}C-NMR (100 MHz, DMSO-d_6, δ/ppm): 194.50, 167.22, 167.19, 166.92, 150.81, 149.24, 147.72, 146.25, 146.15, 144.03, 136.16, 129.05, 128.91, 128.43, 128.22, 128.05, 127.50, 126.62, 126.21, 111.69, 103.64, 63.23, 61.65, 44.46, 43.96, 36.40, 33.62, 18.94, 18.49.

IR (ν/cm^{-1}): 3289, 3043, 3015, 2192, 2181, 2039, 1994, 1611, 1487, 1417, 1350, 1295, 1212, 1128, 1097, 1047, 991, 923, 903, 814, 762, 675.

ESI-MS: observed (expected): 494.1935 (494.1938) [M + Na^+].

3.29 2-(Methacryloyloxy)ethyl 4-(4-hydroxyphenyl)-2-methyl-5-oxo-7-phenyl-1,4,5,6,7,8-hexahydroquinoline-3-carboxylate (M(2)(4))

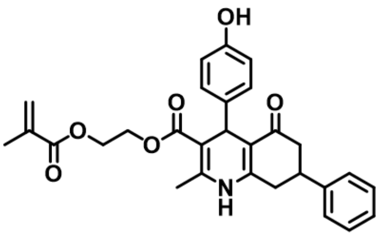

^{1}H-NMR (400 MHz, DMSO-d_6, δ/ppm): 9.20 (s, 1H, OH), 9.10 (s, 1H, CNHC), 6.48-7.35(m, 9H, ph), 6.02 (s, 1H, CH_2=C), 5.70 (s, 1H, CH_2=C), 4.79 (s, 1H, CCHC), 4.16-4.32 (m, 4H, $COOCH_2CH_2$), 2.55-2.85 (m, 2H, C$\underline{H}_2$C=O), 2.27-2.31 (m, 2H, NHCC$\underline{H}_2$), 2.26(s, 3H, C$\underline{H}_3$CNH), 1.87 (s, 3H, C$\underline{H}_3$C=CH_2), 0.80-1.26 (m, 1H, CH_2C$\underline{H}$$CH_2$).

^{13}C-NMR (100 MHz, DMSO-d_6, δ/ppm): 194.17, 167.35, 166.95, 155.93, 155.85, 150.80, 150.44, 145.61, 145.51, 144.07, 138.85, 138.47, 136.16, 129.05, 128.91, 127.50, 126.61, 115.11, 114.91, 112.04, 104.17, 63.26, 61.59, 44.03, 35.34, 35.08, 33.64, 18.90, 18.50.

ESI-MS: observed (expected): 510.1892 (510.1887) [M + Na^+].

IR (ν/cm^{-1}): 2968, 2187, 2051, 2021, 1990, 1660, 1608, 1528, 1380, 1341, 1214, 1127, 1080, 922, 761, 719, 699.

3.30 2-(Methacryloyloxy)ethyl 4-(4-(methoxycarbonyl)phenyl)-2-methyl-5-oxo-7-phenyl-1,4,5,6,7,8-hexahydroquinoline-3-carboxylate (M(3)(4))

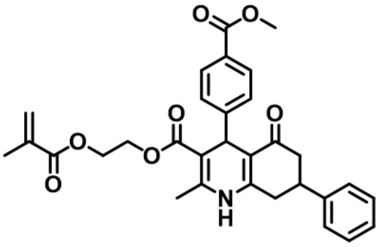

^{1}H-NMR (400 MHz, DMSO-d_6, δ/ppm): 9.27 (s, 1H, CNHC), 7.21-7.77 (m, 9H, ph), 5.94 (s, 1H, $CH_2{=}C$), 5.65 (s, 1H, $CH_2{=}C$), 4.95 (s, 1H, CCHC), 4.15-4.27 (m, 4H, $COOCH_2CH_2$), 3.81 (s, 3H, $COOCH_3$), 2.56-2.84 (m, 2H, $CH_2C{=}O$), 2.32-2.35 (m, 2H, $NHCC\underline{H}_2$), 2.30 (s, 3H, $C\underline{H}_3CNH$), 1.81 (s, 3H, $C\underline{H}_3C{=}CH_2$), 0.80-1.26 (m, 1H, $CH_2C\underline{H}CH_2$).

^{13}C-NMR (100 MHz, DMSO-d_6, δ/ppm): 194.13, 166.90, 166.72, 153.43, 152.96, 151.48, 151.04, 146.84, 143.90, 140.38, 136.09, 129.52, 129.27, 129.05, 128.89, 128.44, 128.34, 127.45, 126.51, 111.15, 110.94, 102.88, 63.18, 61.64, 52.47, 44.36, 43.80, 36.94, 36.68, 18.95, 18.41.

IR (ν/cm^{-1}): 2988, 2194, 2182, 2173, 2153, 2038, 2030, 2004, 1974, 1967, 1661, 1538, 1484, 1380, 1277, 1237, 1198, 1129, 1108, 1077, 814, 797, 738.

ESI-MS: observed (expected): 552.1985 (552.1993) [M + Na^+].

3.31 2-(Methacryloyloxy)ethyl 4-(4-(dimethylamino)phenyl)-2-methyl-5-oxo-7-phenyl-1,4,5,6,7,8-hexahydroquinoline-3-carboxylate (M(4)(4))

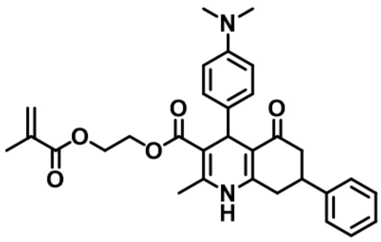

^{1}H-NMR (400 MHz, DMSO-d_6, δ/ppm): 9.08 (s, 1H, CNHC), 6.48-7.37 (m, 9H, ph), 6.02 (s, 1H, $CH_2{=}C$), 5.70 (s, 1H, $CH_2{=}C$), 4.32 (s, 1H, CCHC), 4.17-4.32 (m, 4H, $COOCH_2CH_2$), 2.79 (s, 6H, CH_3NCH_3), 2.53-2.75 (m, 2H, $CH_2C{=}O$), 2.32-2.35 (m, 2H, $NHCC\underline{H}_2$), 2.27 (s, 3H, $C\underline{H}_3CNH$), 1.88 (s, 3H, $C\underline{H}_3C{=}CH_2$), 0.80-1.27 (m, 1H, $CH_2C\underline{H}CH_2$).

^{13}C-NMR (100 MHz, DMSO-d$_6$, δ/ppm): 194.49, 194.18, 190.42, 167.41, 166.95, 150.29, 149.25, 145.33, 144.08, 136.59, 136.30, 136.17, 128.94, 128.55, 128.41, 127.50, 126.53, 112.76, 112.17, 111.60, 104.25, 63.29, 61.56, 44.13, 44.12, 35.22, 34.95, 34.06, 33.70, 18.89, 18.51.

IR (ν/cm^{-1}): 3675, 2934, 2901, 2213, 2180, 2172, 2166, 2041, 2014, 1993, 1984, 1974, 1660, 1607, 1539, 1482, 1423, 1380, 1190, 1163, 1126, 944, 792, 720.

ESI-MS: observed (expected): 537.2357 (537.2360) [M + Na$^+$].

3.32 2-(Methacryloyloxy)ethyl (E)-2-methyl-5-oxo-7-phenyl-4-(4-styrylphenyl)-1,4,5,6,7,8-hexahydroquinoline-3-carboxylate (M(5)(4))

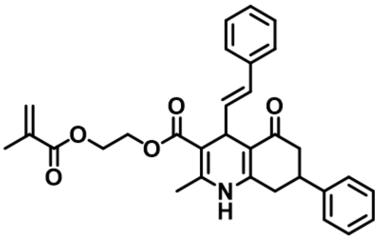

^{1}H-NMR (400 MHz, DMSO-d$_6$, δ/ppm): 9.17 (s, 1H, CNHC), 7.12-7.41 (m, 10H, ph), 6.10 (s, 1H, phC$\underline{H}$=CH), 6.01 (s, 1H, phCH=C$\underline{H}$), 6.00 (s, 1H, CH_2=C), 5.62 (s, 1H, CH_2=C), 4.58 (s, 1H, CCHC), 4.21-4.43 (m, 4H, $COOCH_2CH_2$), 2.58-2.90 (m, 2H, CH_2C=O), 2.27-2.30 (m, 2H, NHCC$\underline{H}_2$), 2.25 (s, 3H, C$\underline{H}_3$CNH), 1.83 (s, 3H, C$\underline{H}_3$C=CH_2), 0.79-1.23 (m, 1H, CH_2C$\underline{H}$$CH_2$).

^{13}C-NMR (100 MHz, DMSO-d$_6$, δ/ppm): 194.22, 166.94, 166.94, 151.98, 151.56, 147.11, 147.11, 144.06, 144.05, 137.64, 136.16, 136.16, 128.99, 128.99, 128.20, 127.97, 127.52, 127.08, 126.43, 126.40, 109.58, 101.18, 99.42, 63.26, 63.20, 33.30, 33.88, 33.16, 18.98, 18.45, 18.44.

IR (ν/cm^{-1}): 3664, 2212, 2195, 2188, 2155, 2026, 2006, 1958, 1716, 1659, 1460, 1450,

1212, 1189, 827, 813, 720, 697.

ESI-MS: observed (expected): 520.2094 (520.2094) [M + Na^+].

3.33 2-(Methacryloyloxy)ethyl 4-(4-hydroxy-3-methoxyphenyl)-2-methyl-5-oxo-7-phenyl-1,4,5,6,7,8-hexahydroquinoline-3-carboxylate (M(6)(4))

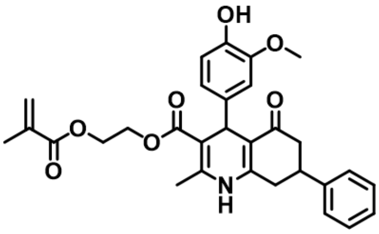

^{1}H-NMR (400 MHz, DMSO-d_6, δ/ppm): 9.10 (s, 1H, OH), 8.64 (s, 1H, CNHC), 6.45-7.39 (m, 8H, ph), 5.99 (s, 1H, CH_2=C), 5.68 (s, 1H, CH_2=C), 4.81 (s, 1H, CCHC), 4.21-4.32 (m, 4H, $COOCH_2CH_2$), 3.68 (s, 3H, OCH_3), 2.58-2.90 (m, 2H, CH_2C=O), 2.27-2.30 (m, 2H, $NHCC\underline{H}_2$), 2.27 (s, 3H, $C\underline{H}_3$CNH), 1.85 (s, 3H, $C\underline{H}_3C=CH_2$), 0.83-1.23 (m, 1H, $CH_2C\underline{H}CH_2$).

^{13}C-NMR (100 MHz, DMSO-d_6, δ/ppm): 194.58, 194.27, 167.42, 166.94, 153.57, 150.80, 147.31, 145.15, 144.06, 139.42, 136.14, 129.04, 128.88, 127.51, 126.59, 119.97, 115.92, 115.60, 112.46, 112.05, 104.15, 63.19, 62.84, 61.58, 56.11, 55.97, 49.99, 30.54, 18.89, 18.46.

IR (ν/cm^{-1}): 3675, 2988, 2214, 2205, 2168, 2044, 2029, 2009, 1715, 1660, 1538, 1481, 1440, 1380, 1213, 1138, 1076, 838, 715, 699.

ESI-MS: observed (expected): 540.1993 (540.1990) [M + Na^+].

3.34 2-(Methacryloyloxy)ethyl 4-hexyl-2-methyl-5-oxo-7-phenyl-1,4,5,6,7,8-hexahydroquinoline-3-carboxylate (M(7)(4))

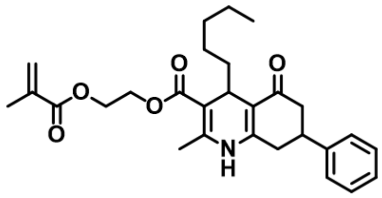

^{1}H-NMR (400 MHz, DMSO-d_6, δ/ppm): 8.94 (s, 1H, CNHC), 7.20-7.38 (m, 5H, ph), 6.03 (s, 1H, CH_2=C), 5.69 (s, 1H, CH_2=C), 4.19-4.35 (m, 4H, $COOCH_2CH_2$), 3.82 (s, 1H, CCHC), 2.40-2.79 (m, 2H, CH_2C=O), 2.17-2.28 (m, 2H, $NHCC\underline{H}_2$), 2.28 (s, 3H, $C\underline{H}_3$CNH), 1.87 (s, 3H, $C\underline{H}_3C=CH_2$), 0.92-1.36 (m, 9H, $CH_2C\underline{H}CH_2$, $(C\underline{H}_2)_4CH_3$), 0.93-0.95 (m, 3H, $CH_2C\underline{H}_3$).

^{13}C-NMR (100 MHz, DMSO-d_6, δ/ppm): 194.49, 177.73, 167.46, 166.90, 152.05, 151.45, 146.88, 144.14, 143.95, 136.22, 129.06, 128.84, 127.51, 127.46, 126.93, 63.27, 61.46, 56.54, 36.24, 33.38, 32.24, 29.95, 24.38, 22.68, 18.83, 18.46, 14.59, 14.50.

IR (ν/cm^{-1}): 3663, 3290, 3045, 2942, 2171, 2155, 2041, 2026, 2003, 1975, 1709, 1661, 1480, 1453, 1355, 1307, 1211, 1157, 1060, 1018, 943, 911, 833, 801, 699.

ESI-MS: observed (expected): 488.2405 (488.2407) [M + Na^+].

3.35 2-(Methacryloyloxy)ethyl-2-methyl-4-ferrocenyl-5-oxo-7-phenyl-1,4,5,6,7,8-hexahydroquinoline-3-carboxylate (M(8)(4))

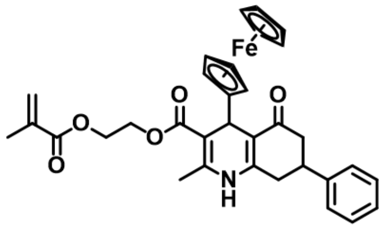

^{1}H-NMR (400 MHz, DMSO-d_6, δ/ppm): 9.21 (s, 1H, CNHC), 7.23-7.41 (m, 5H, ph), 6.09 (s, 1H, CH_2=C), 5.74 (s, 1H, CH_2=C), 4.72 (s, 1H, CCHC), 4.34-4.44 (m, 4H, $COOCH_2CH_2$), 3.76-3.96 (m, 9H, Cp), 2.56-2.93 (m, 2H, CH_2C=O), 2.26-2.29 (m, 2H, $NHCC\underline{H}_2$), 2.24 (s, 3H, $C\underline{H}_3$CNH), 1.93 (s, 3H, $C\underline{H}_3C=CH_2$), 0.92-1.36 (m, 1H,

$CH_2C\underline{H}CH_2$).

^{13}C-NMR (100 MHz, DMSO-d_6, δ/ppm): 194.30, 167.89, 167.71, 167.01, 166.20, 151.16, 145.55, 144.05, 143.76, 136.20, 129.09, 128.97, 127.60, 127.51, 127.07, 126.71, 126.33, 111.15, 103.80, 96.44, 68.88, 68.87, 68.86, 66.86, 66.70, 66.56, 66.45, 66.24, 63.39, 61.93, 44.04, 28.55, 18.54.

IR (ν/cm^{-1}): 3663, 3289, 2932, 2212, 2046, 2019, 1945, 1704, 1598, 1485, 1388, 1348, 1222, 1170, 1097, 1061, 892, 816, 752, 732.

ESI-MS: observed (expected): 602.1601 (602.1604) [M + Na$^+$].

3.36 2-(Methacryloyloxy)ethyl-2-methyl-4-(4-nitrophenyl)-5-oxo-7-phenyl-1,4,5,6,7,8-hexahydroquinoline-3-carboxylate (M(9)(4))

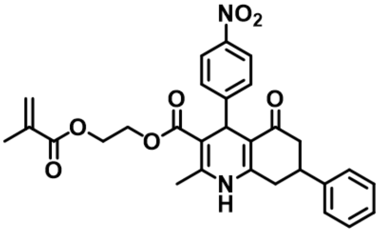

^{1}H-NMR (400 MHz, DMSO-d_6, δ/ppm): 9.36 (s, 1H, CNHC), 7.19-8.07 (m, 9H, ph), 5.90 (s, 1H, CH_2=C), 5.62 (s, 1H, CH_2=C), 5.00 (s, 1H, CCHC), 4.04-4.28 (m, 4H, $COOCH_2CH_2$), 2.56-2.87 (m, 2H, CH_2C=O), 2.26-2.29 (m, 2H, $NHCC\underline{H}_2$), 2.24 (s, 3H, $C\underline{H}_3$CNH), 1.92 (s, 3H, $C\underline{H}_3C=CH_2$), 0.92-1.35 (m, 1H, $CH_2C\underline{H}CH_2$).

^{13}C-NMR (100 MHz, DMSO-d_6, δ/ppm): 194.52, 194.15, 166.77, 166.69, 155.08, 151.26, 147.48, 147.41, 146.19, 146.09, 143.77, 143.67, 136.07, 129.31, 128.88, 127.42, 126.45, 123.80, 123.53, 110.82, 102.37, 63.13, 61.69, 57.11, 43.70, 38.94, 37.17, 18.97, 18.35.

IR (ν/cm^{-1}): 3617, 2972, 2154, 2151, 2037, 1997, 1990, 1975, 1959, 1952, 1549, 1515,

1484, 1381, 1360, 1266, 1196, 1166, 1078, 856, 791, 740, 699.

ESI-MS: observed (expected): 539.1789 (539.1788) [M + Na^+].

3.37 2-(Methacryloyloxy)ethyl 2,6,6-trimethyl-5-oxo-4-phenyl-1,4,5,6,7,8-hexahydroquinoline-3-carboxylate (M(1)(5))

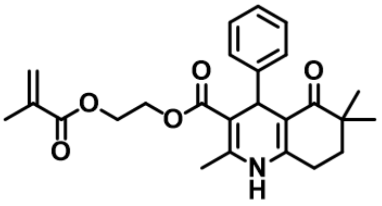

^{1}H-NMR (400 MHz, DMSO-d_6, δ/ppm): 9.15 (s, 1H, CNHC), 7.02-7.15 (m, 5H, ph), 6.01 (s, 1H, CH_2=C), 5.70 (s, 1H, CH_2=C), 4.86 (s, 1H, CCHC), 4.18-4.28 (m, 4H, $COOCH_2CH_2$), 2.26 (s, 3H, $NHCC\underline{H}_3$), 1.87 (s, 3H, $C\underline{H}_3C{=}CH_2$), 1.65-1.76 (m, 4H, CCH_2CH_2C), 0.98 (s, 3H, $C\underline{H}_3CCH_3$), 0.88 (s, 3H, $CH_3CC\underline{H}_3$).

^{13}C-NMR (100 MHz, DMSO-d_6, δ/ppm): 199.99, 167.29, 166.93, 150.15, 148.25, 146.21, 136.16, 129.80, 128.32, 127.74, 126.61, 126.16, 126.14, 110.84, 109.87, 103.21, 63.28, 61.58, 36.30, 34.67, 25.64, 24.66, 23.41, 18.91, 18.48.

IR (ν/cm^{-1}): 3301, 2966, 2181, 2172, 2036, 2016, 2010, 1719, 1703, 1664, 1532, 1415, 1380, 1270, 1257, 1231, 1120, 1073, 951, 857, 797, 730, 697.

ESI-MS: observed (expected): 446.1935 (446.1938) [M + Na^+].

3.38 2-(Methacryloyloxy)ethyl 4-(4-hydroxyphenyl)-2,6,6-trimethyl-5-oxo-1,4,5,6,7,8-hexahydroquinoline-3-carboxylate (M(2)(5))

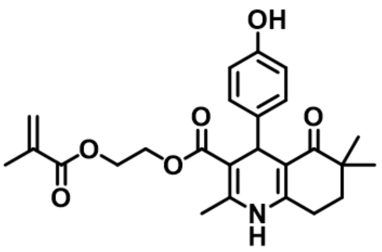

^{1}H-NMR (400 MHz, DMSO-d_6, δ/ppm): 9.09 (s, 1H, CNHC), 6.50-6.88 (m, 4H, ph),

6.02 (s, 1H, $CH_2{=}C$), 5.70 (s, 1H, $CH_2{=}C$), 4.74 (s, 1H, CCHC), 4.16-4.30 (m, 4H, $COOCH_2CH_2$), 2.23 (s, 3H, $NHCC\underline{H}_3$), 1.89 (s, 3H, $C\underline{H}_3C{=}CH_2$), 1.64-1.78 (m, 4H, CCH_2CH_2C), 0.97 (s, 3H, $C\underline{H}_3CCH_3$), 0.88 (s, 3H, $CH_3CC\underline{H}_3$).

^{13}C-NMR (100 MHz, DMSO-d_6, δ/ppm): 200.00, 167.44, 166.96, 155.78, 149.76, 145.59, 138.95, 136.16, 128.62, 126.61, 116.39, 115.03, 111.42, 110.20, 109.75, 103.76, 63.32, 61.52, 35.24, 34.72, 25.68, 24.68, 23.40, 18.88, 18.50.

IR (ν/cm^{-1}): 3302, 2972, 2188, 2168, 2132, 2011, 1976, 1713, 1609, 1510, 1413, 1346, 1272, 1247, 1210, 1178, 1140, 1088, 973, 918, 853, 837, 792, 769.

ESI-MS: observed (expected): 462.1887 (462.1887) [M + Na$^+$].

3.39 2-(Methacryloyloxy)ethyl 4-(4-(methoxycarbonyl)phenyl)-2,6,6-trimethyl-5-oxo-1,4,5,6,7,8-hexahydroquinoline-3-carboxylate (M(3)(5))

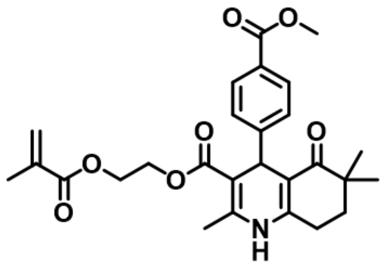

^{1}H-NMR (400 MHz, DMSO-d_6, δ/ppm): 9.23 (s, 1H, CNHC), 7.24-7.75 (m, 4H, ph), 5.95 (s, 1H, $CH_2{=}C$), 5.65 (s, 1H, $CH_2{=}C$), 4.92 (s, 1H, CCHC), 4.13-4.26 (m, 4H, $COOCH_2CH_2$), 3.80 (s, 3H, OCH_3), 2.29 (s, 3H, $NHCC\underline{H}_3$), 1.83 (s, 3H, $C\underline{H}_3C{=}CH_2$), 1.63-1.76 (m, 4H, CCH_2CH_2C), 0.97 (s, 3H, $C\underline{H}_3CCH_3$), 0.86 (s, 3H, $CH_3CC\underline{H}_3$).

^{13}C-NMR (100 MHz, DMSO-d_6, δ/ppm): 199.97, 169.24, 166.99, 166.71, 153.60, 150.42, 148.34, 146.90, 136.24, 136.10, 129.40, 128.18, 127.63, 126.51, 109.43, 103.68, 102.41, 63.24, 61.57, 52.43, 36.91, 34.59, 25.52, 24.62, 23.42, 18.93, 18.41.

IR (ν/cm^{-1}): 3301, 2960, 2147, 2032, 2021, 2001, 1978, 1970, 1717, 1661, 1532, 1444,

1435, 1379, 1275, 1228, 1133, 1084, 1073, 1019, 952, 917, 793, 767, 711.

ESI-MS: observed (expected): 504.1989 (504.1993) [M + Na^+].

3.40 2-(Methacryloyloxy)ethyl 4-(4-(dimethylamino)phenyl)-2,6,6-trimethyl-5-oxo-1,4,5,6,7,8-hexahydroquinoline-3-carboxylate (M(4)(5))

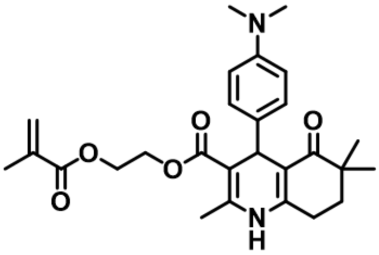

^{1}H-NMR (400 MHz, DMSO-d_6, δ/ppm): 9.05 (s, 1H, CNHC), 6.48-6.93 (m, 4H, ph), 6.02 (s, 1H, CH_2=C), 5.70 (s, 1H, CH_2=C), 4.73 (s, 1H, CCHC), 4.17-4.27 (m, 4H, $COOCH_2CH_2$), 2.78 (s, 6H, CH_3NCH_3), 2.23 (s, 3H, NHCC$\underline{H}_3$), 1.87 (s, 3H, C$\underline{H}_3$C=CH_2), 1.67-1.75 (m, 4H, CCH_2CH_2C), 0.97 (s, 3H, C$\underline{H}_3$$CCH_3$), 0.89 (s, 3H, CH_3CC$\underline{H}_3$).

^{13}C-NMR (100 MHz, DMSO-d_6, δ/ppm): 200.15, 167.51, 166.96, 149.65, 149.14, 147.14, 145.40, 136.63, 136.17, 128.23, 126.55, 112.72, 112.65, 111.33, 110.24, 103.88, 61.54, 49.73, 45.01, 35.05, 34.76, 25.79, 24.72, 23.42, 18.87, 18.51, 18.51.

IR (ν/cm^{-1}): 3675, 3303, 2936, 2210, 2172, 2155, 2017, 1983, 1702, 1597, 1505, 1422, 1242, 1189, 1132, 1061, 953, 891, 793, 759.

ESI-MS: observed (expected): 489.2363 (489.2360) [M + Na^+].

3.41 2-(Methacryloyloxy)ethyl (E)-2,6,6-trimethyl-5-oxo-4-(4-styrylphenyl)-1,4,5,6,7,8-hexahydroquinoline-3-carboxylate (M(5)(5))

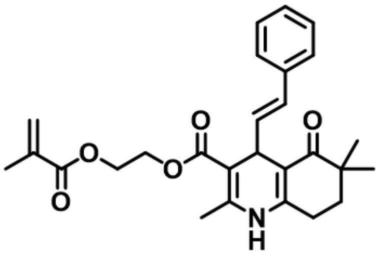

^{1}H-NMR (400 MHz, DMSO-d_6, δ/ppm): 9.13 (s, 1H, CNHC), 7.15-7.25 (m, 5H, ph), 6.03 (s, 1H, phC$\underline{H}$=CH), 6.02 (s, 1H, phCH=C$\underline{H}$), 6.01 (s, 1H, CH_2=C), 5.63 (s, 1H, CH_2=C), 4.46 (s, 1H, CCHC), 4.19-4.36 (m, 4H, $COOCH_2CH_2$), 2.26 (s, 3H, NHCC$\underline{H}_3$), 1.83 (s, 3H, C$\underline{H}_3$C=CH_2), 1.73-1.77 (m, 4H, CCH_2CH_2C), 1.04 (s, 3H, C$\underline{H}_3$$CCH_3$), 1.00 (s, 3H, CH_3CC$\underline{H}_3$).

^{13}C-NMR (100 MHz, DMSO-d_6, δ/ppm): 200.03, 167.17, 150.94, 147.52, 147.14, 137.67, 136.15, 132.98, 128.98, 127.47, 126.60, 126.45, 108.75, 107.51, 100.76, 99.42, 63.29, 61.62, 34.87, 33.21, 25.90, 24.69, 23.48, 18.93, 18.47, 18.44, 18.38.

IR (ν/cm^{-1}): 3662, 3300, 2940, 2212, 2160, 2010, 1966, 1707, 1625, 1598, 1461, 1416, 1317, 1289, 1233, 1180, 1099, 1076, 1014, 987, 952, 939, 828, 795, 749, 728.

ESI-MS: observed (expected): 472.2093 (472.2094) [M + Na^+].

3.42 2-(Methacryloyloxy)ethyl 4-(4-hydroxy-3-methoxyphenyl)-2,6,6-trimethyl-5-oxo-1,4,5,6,7,8-hexahydroquinoline-3-carboxylate (M(6)(5))

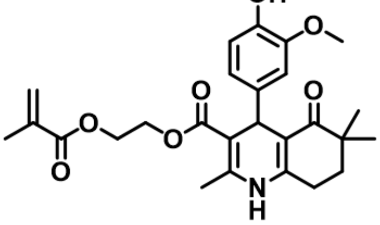

^{1}H-NMR (400 MHz, DMSO-d_6, δ/ppm): 9.08 (s, 1H, OH), 8.61 (s, 1H, CNHC), 6.46-6.66 (m, 3H, ph), 6.00 (s, 1H, CH_2=C), 5.68 (s, 1H, CH_2=C), 4.78 (s, 1H, CCHC), 4.18-4.29 (m, 4H, $COOCH_2CH_2$), 3.64 (s, 3H, OCH_3), 2.24 (s, 3H, NHCC$\underline{H}_3$), 1.86 (s, 3H,

$C\underline{H}_3C=CH_2$), 1.69-1.76 (m, 4H, CCH_2CH_2C), 0.98 (s, 3H, $C\underline{H}_3CCH_3$), 0.92 (s, 3H, $CH_3CC\underline{H}_3$).

^{13}C-NMR (100 MHz, DMSO-d_6, δ/ppm): 200.14, 167.50, 166.95, 149.89, 147.36, 145.67, 145.08, 139.45, 136.14, 126.59, 119.73, 115.49, 112.16, 111.18, 110.12, 103.52, 63.26, 61.54, 55.92, 35.37, 34.80, 25.74, 24.68, 23.40, 18.89, 18.46.

IR (ν/cm^{-1}): 3675, 3576, 2988, 2938, 2239, 2182, 2172, 2161, 2017, 1998, 1979, 1976, 1951, 1608, 1559, 1381, 1194, 1140, 1066, 959, 835, 779.

ESI-MS: observed (expected): 492.1997 (492.1993) [M + Na^+].

3.43 2-(Methacryloyloxy)ethyl 4-hexyl-2,6,6-trimethyl-5-oxo-1,4,5,6,7,8-hexahydroquinoline-3-carboxylate (M(7)(5))

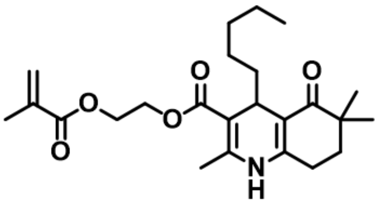

^{1}H-NMR (400 MHz, DMSO-d_6, δ/ppm): 8.90 (s, 1H, CNHC), 6.04 (s, 1H, $CH_2=C$), 5.70 (s, 1H, $CH_2=C$), 4.18-4.39 (m, 4H, $COOCH_2CH_2$), 3.78 (s, 1H, CCHC), 2.20 (s, 3H, $NHCC\underline{H}_3$), 1.88 (s, 3H, $C\underline{H}_3C=CH_2$), 1.70-1.76 (m, 4H, CCH_2CH_2C), 0.93-1.20 (m, 8H, $(C\underline{H}_2)_4CH_3$), 0.98 (s, 3H, $C\underline{H}_3CCH_3$), 0.92 (s, 3H, $CH_3CC\underline{H}_3$), 0.77 (m, 3H, $CH_2C\underline{H}_3$).

^{13}C-NMR (100 MHz, DMSO-d_6, δ/ppm): 200.44, 167.56, 166.91, 150.88, 146.83, 136.22, 112.90, 110.33, 109.22, 102.47, 66.44, 63.29, 61.38, 36.46, 35.04, 34.91, 32.01, 29.85, 26.17, 24.19, 23.49, 22.55, 18.44, 14.35.

IR (ν/cm^{-1}): 3675, 3302, 2939, 2219, 2162, 2127, 1993, 1968, 1697, 1598, 1432, 1342, 1228, 1192, 1138, 1070, 1057, 953, 825, 752.

ESI-MS: observed (expected): 440.2407 (440.2407) [M + Na $^{+}$].

3.44 2-(Methacryloyloxy)ethyl -2,6,6-trimethyl-4-ferrocenyl -5-oxo-1,4,5,6,7,8-hexahydroquinoline-3-carboxylate (M(8)(5))

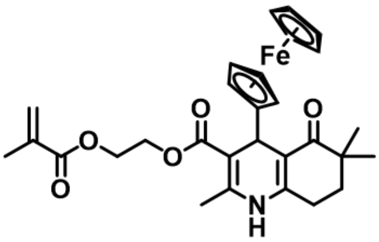

^{1}H-NMR (400 MHz, DMSO-d$_6$, δ/ppm): 9.22 (s, 1H, CNHC), 6.08 (s, 1H, CH_2=C), 5.74 (s, 1H, CH_2=C), 4.69 (s, 1H, CCHC), 4.31-4.39 (m, 4H, $COOCH_2CH_2$), 3.69-3.99 (m, 9H, Cp), 2.21 (s, 3H, NHCC$\underline{H}_3$), 1.91 (s, 3H, C$\underline{H}_3$C=CH_2), 1.74-1.84 (m, 4H, CCH_2CH_2C), 1.11 (s, 3H, C$\underline{H}_3$$CCH_3$), 1.01 (s, 3H, CH_3CC$\underline{H}_3$).

^{13}C-NMR (100 MHz, DMSO-d$_6$, δ/ppm): 200.21, 167.70, 167.02, 150.88, 145.79, 136.20, 126.68, 108.82, 103.90, 96.65, 69.01, 68.84, 68.69, 66.91, 66.66, 66.38, 65.96, 63.42, 61.79, 34.92, 28.40, 26.36, 26.14, 24.51, 23.63, 23.52, 18.78, 18.52, 18.44.

IR (ν/cm^{-1}): 3675, 3293, 2972, 2935, 2187, 2157, 2140, 1992, 1977, 1958, 1710, 1668, 1601, 1481, 1379, 1270, 1204, 1191, 1162, 1074, 1162, 1074, 969, 921, 826, 754, 723.

ESI-MS: observed (expected): 554.1598 (554.1601) [M + Na^{+}].

3.45 2-(Methacryloyloxy)ethyl 2,6,6-trimethyl-4-(4-nitrophenyl)-5-oxo-1,4,5,6,7,8-hexahydroquinoline-3-carboxylate (M(9)(5))

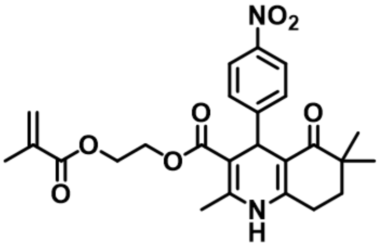

^{1}H-NMR (400 MHz, DMSO-d$_6$, δ/ppm): 9.32 (s, 1H, CNHC), 7.39-8.01 (m, 4H, ph),

5.91 (s, 1H, CH_2=C), 5.63 (s, 1H, CH_2=C), 4.96 (s, 1H, CCHC), 4.14-4.32 (m, 4H, $COOCH_2CH_2$), 2.31 (s, 3H, NHCC$\underline{H}_3$), 1.80 (s, 3H, C$\underline{H}_3$C=CH_2), 1.63-1.75 (m, 4H, CCH_2CH_2C), 0.98 (s, 3H, C$\underline{H}_3$CCH$_3$), 0.86 (s, 3H, CH_3CC$\underline{H}_3$).

^{13}C-NMR (100 MHz, DMSO-d_6, δ/ppm): 200.00, 166.79, 155.75, 150.71, 149.08, 147.44, 146.09, 136.08, 130.31, 129.08, 126.45, 123.69, 109.09, 101.92, 100.21, 97.60, 63.18, 61.63, 37.19, 34.50, 25.49, 24.59, 23.43, 18.95, 18.36.

IR (ν/cm^{-1}): 3663, 2971, 2194, 2186, 2168, 2157, 2147, 2023, 2010, 1986, 1968, 1663, 1604, 1515, 1484, 1421, 1305, 1189, 1066, 942, 919, 842, 793, 756, 704.

ESI-MS: observed (expected): 491.1783 (491.1789) [M + Na$^+$].

3.46 Copolymerization of M(X)(Y) and PEGMA

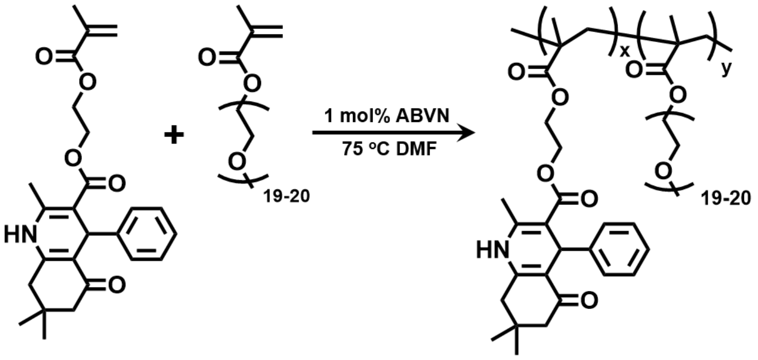

As a typical example, M(1)(1) (212 mg, 0.5 mmol), PEGMA-950 (475 mg, 0.5 mmol), ABVN (3 mg, 0.01 mmol) were charged into a centrifuge tube along with DMF (1.0 mL). The tube was sealed with a rubber septum and purged by nitrogen flow for 15 min, then kept in a 75°C isothermal shaker for 12 h. At the end of the polymerization, the mixture was precipitated in diethyl ether 3 times, then dried under vacuum to obtain a viscous yellowish copolymer P(1)(1) (591 mg, yield: 86%).

All other copolymers P(X)(Y) were parallelly prepared through the same procedure.

3.47 Poly(poly (ethylene glycol) methyl ether methacrylate) (P(PEGMA))

PEGMA-950 (950 mg, 1 mmol), ABVN (3 mg, 0.01 mmol) were charged into a dry Schlenk tube along with DMF (1.0 mL). The Schlenk tube was sealed with a rubber septum and purged by nitrogen flow for 15 min, then put into a 75°C oil bath for 12 h. At the end of the polymerization, the mixture was precipitated in diethyl ether 3 times, then dried under vacuum to obtain P(PEGMA) as a white powder (855 mg, yield: 90%).

Supporting Data

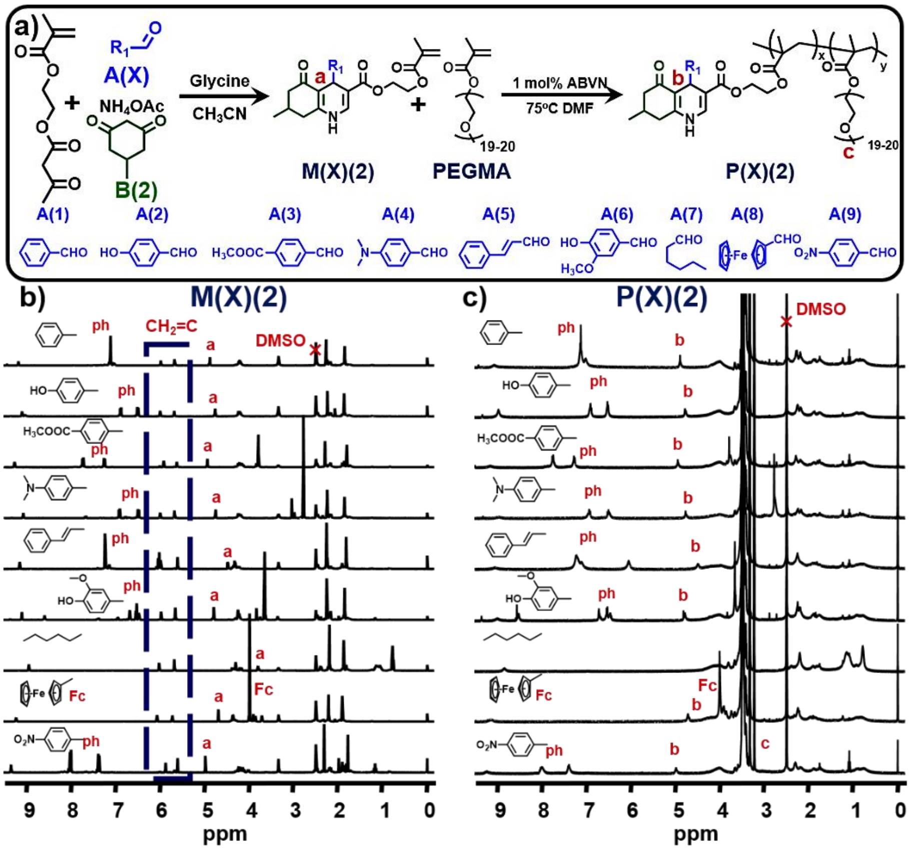

Figure S1: M(X)(2) and P(X)(2). (a) Preparation of M(X)(2) via Hantzsch's reaction and P(X)(2) via free radical polymerisation. (b) ^{1}H NMR spectra (DMSO-d_6, 400M) of M(X)(2). (c) ^{1}H NMR spectra (DMSO-d_6, 400M) of P(X)(2).

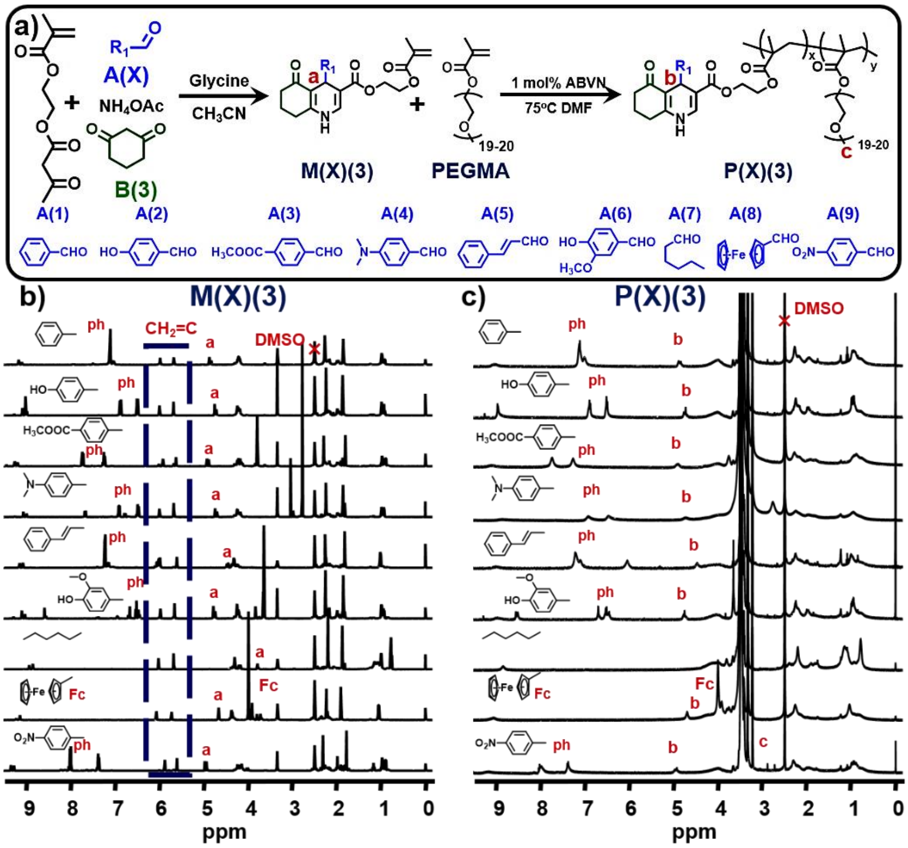

Figure S2: M(X)(3) and P(X)(3). (a) Preparation of M(X)(3) via Hantzsch's reaction and P(X)(3) via free radical polymerisation. (b) ^{1}H NMR spectra (DMSO-d_6, 400M) of M(X)(3). (c) ^{1}H NMR spectra (DMSO-d_6, 400M) of P(X)(3).

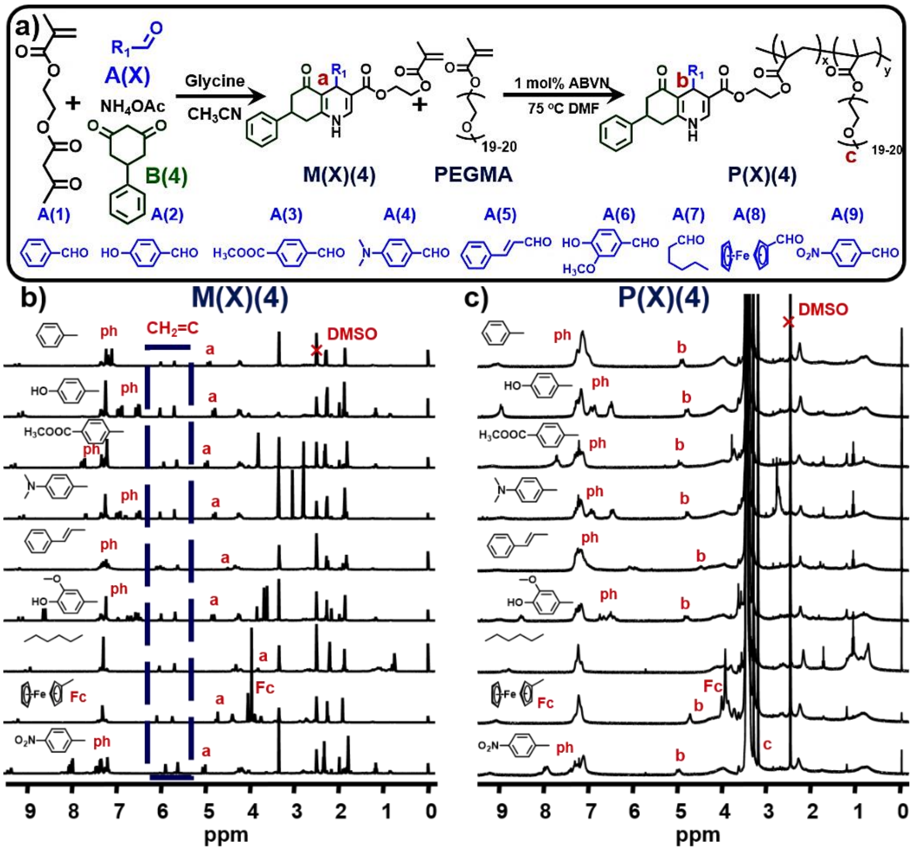

Figure S3: M(X)(4) and P(X)(4). (a) Preparation of M(X)(4) via Hantzsch's reaction and P(X)(4) via free radical polymerisation. (b) ^{1}H NMR spectra (DMSO-d_6, 400M) of M(X)(4). (c) ^{1}H NMR spectra (DMSO-d_6, 400M) of P(X)(4).

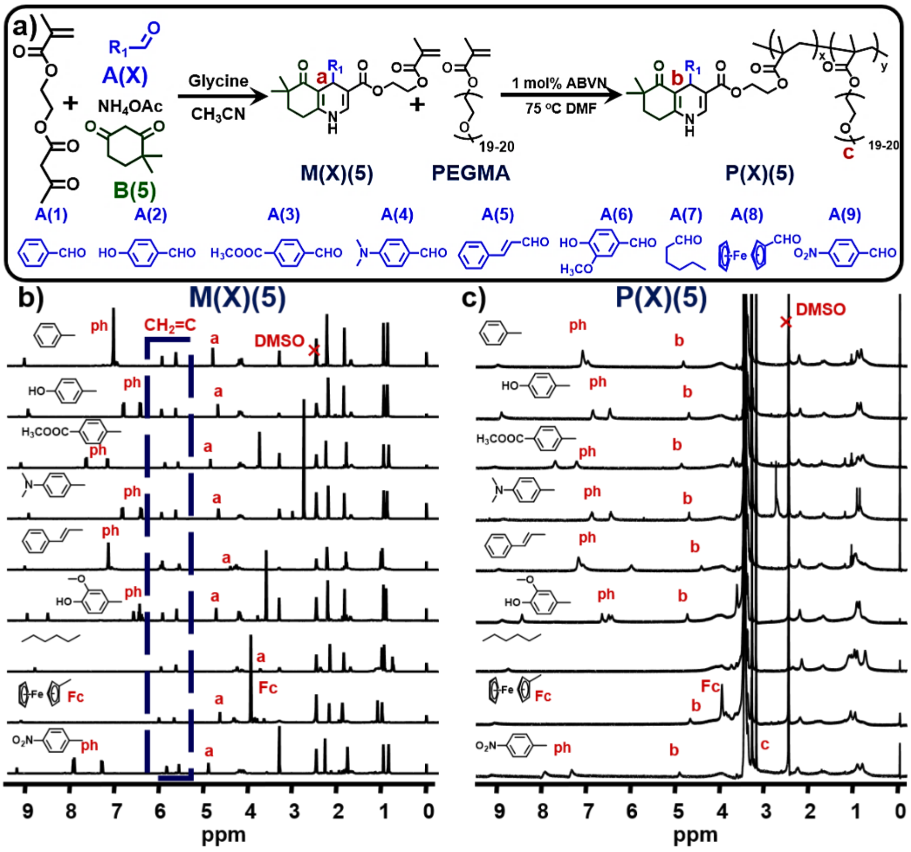

Figure S4: M(X)(5) and P(X)(5). (a) Preparation of M(X)(5) via Hantzsch's reaction and P(X)(5) via free radical polymerisation. (b) ^{1}H NMR spectra (DMSO-d_6, 400M) of M(X)(5). (c) ^{1}H NMR spectra (DMSO-d_6, 400M) of P(X)(5).

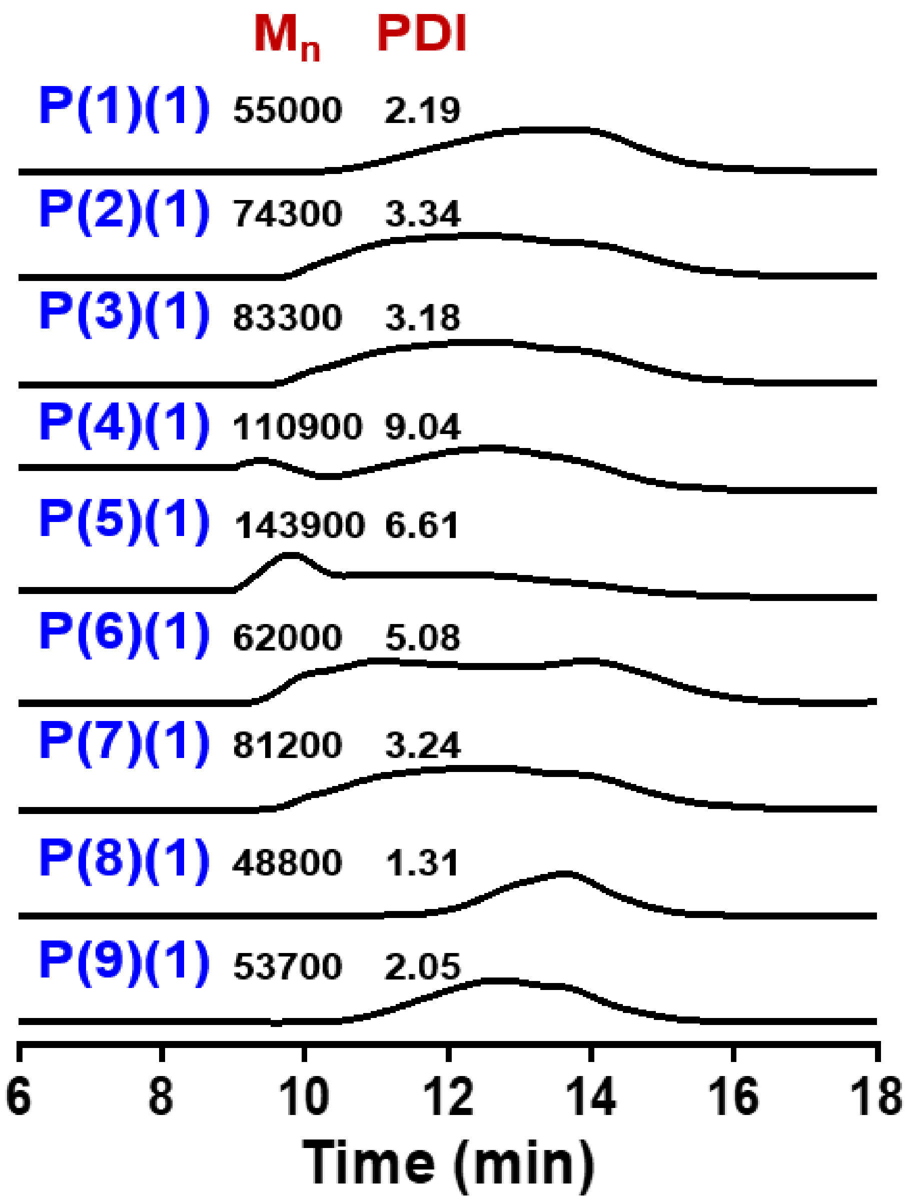

Figure S5: GPC traces of P(X)(1).

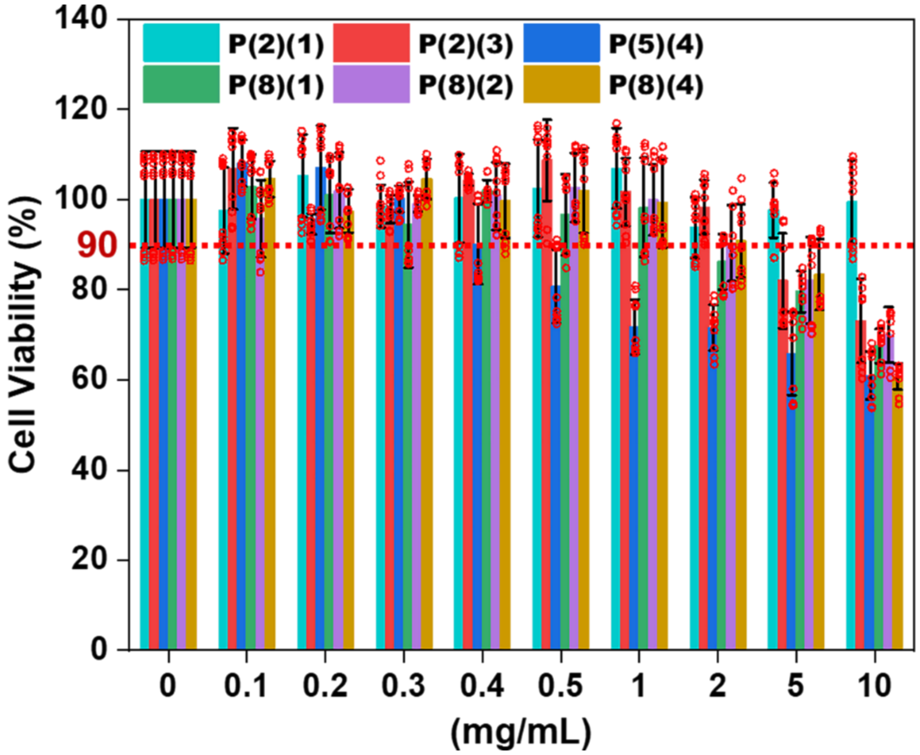

Figure S6: Cytotoxicity of polymers to L929 cells, 48 h culture. Cell viability in a culture medium only was defined as 100% viability. Data are presented as mean values ± SD (n = 10 independent experiments). Source data are provided as a Source Data file.

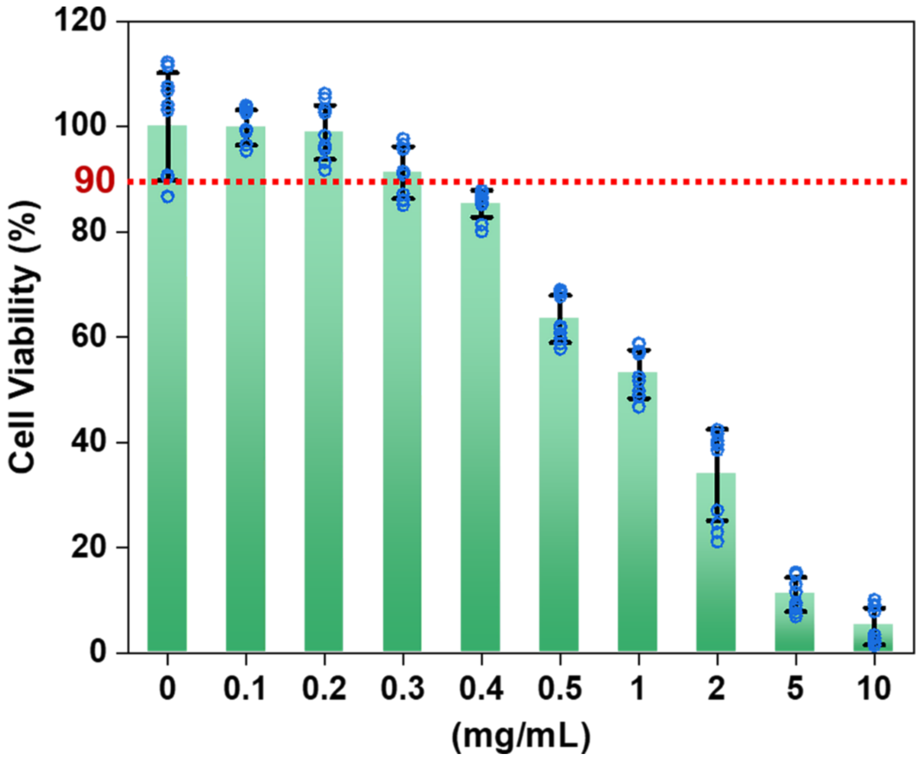

Figure S7: Cytotoxicity of amifostine to L929 cells, 48 h culture. Cell viability in a culture medium only was defined as 100% viability. Data are presented as mean values ± SD (n = 10 independent experiments). Source data are provided as a Source Data file.

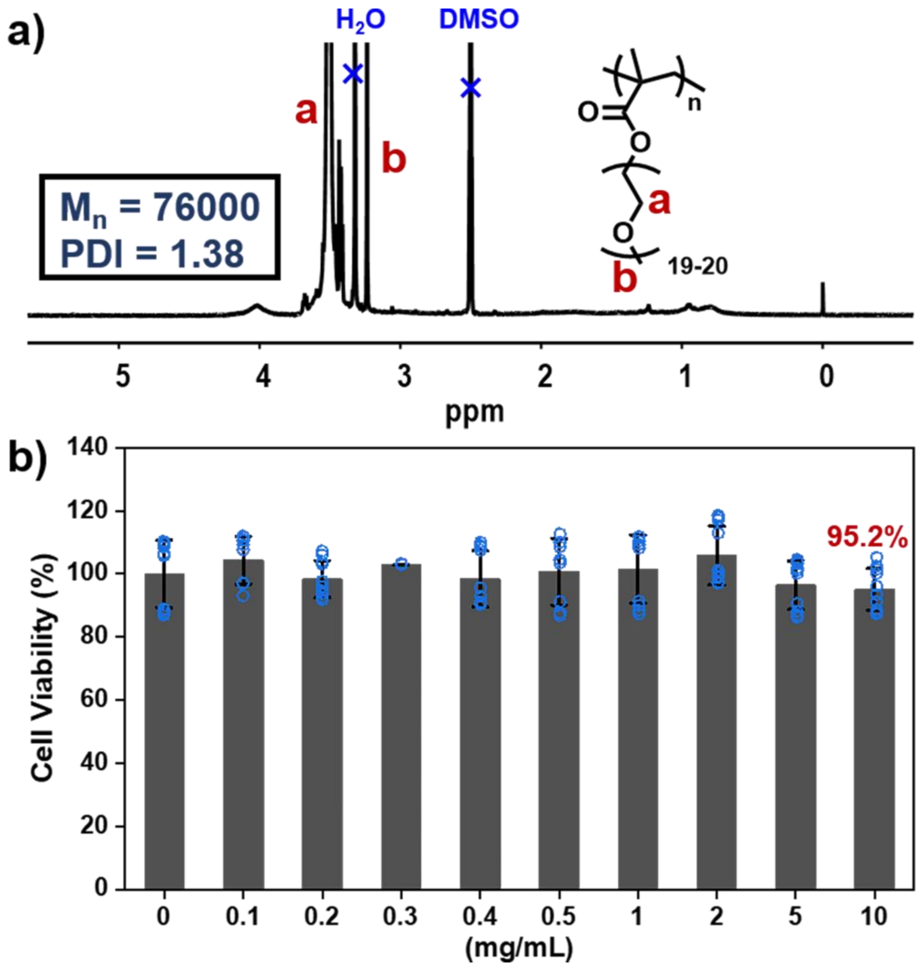

Figure S8: P(PEGMA) and its cytotoxicity to L929 cells. (a) ^{1}H NMR spectra (DMSO-d_6, 400M) of P(PEGMA). (b) Cytotoxicity of P(PEGMA) to L929 cells, 48 h culture. Cell viability in a culture medium only was defined as 100% viability. Data are presented as mean values ± SD (n = 10 independent experiments). Source data are provided as a Source Data file.

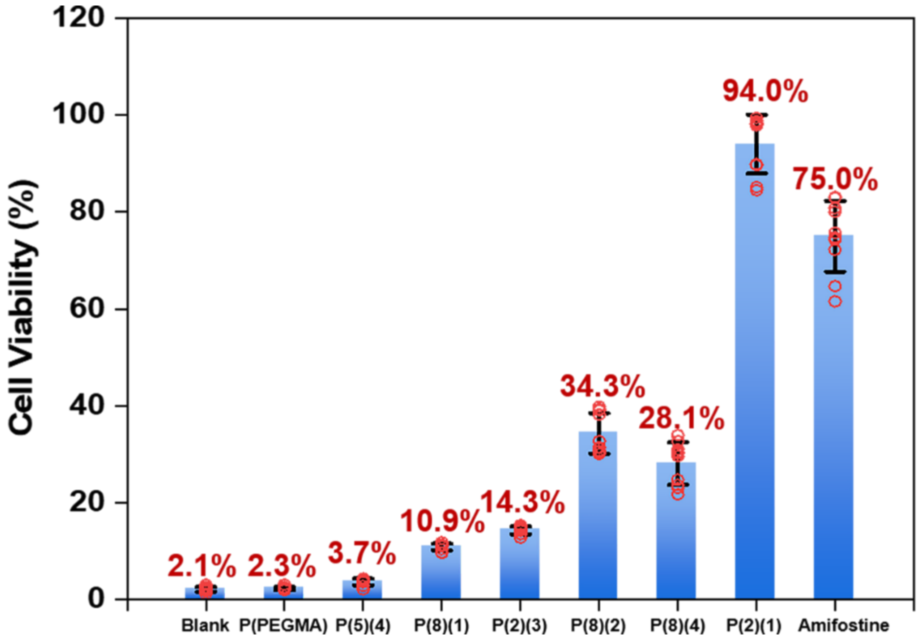

Figure S9: Viability of L929 cells. Cells were exposed to 80 Gy X-ray radiation under different culture conditions, 48 h culture: medium only (blank), P(PEGMA) (10 mg/mL), P(5)(4) (0.4 mg/mL), P(8)(1) (1 mg/mL), P(2)(3) (2 mg/mL), P(8)(2) (2 mg/mL), P(8)(4) (2 mg/mL), P(2)(1) (10 mg/mL) and amifostine (0.3 mg/mL). Data are presented as mean values ± SD (n = 10 independent experiments). Source data are provided as a Source Data file.

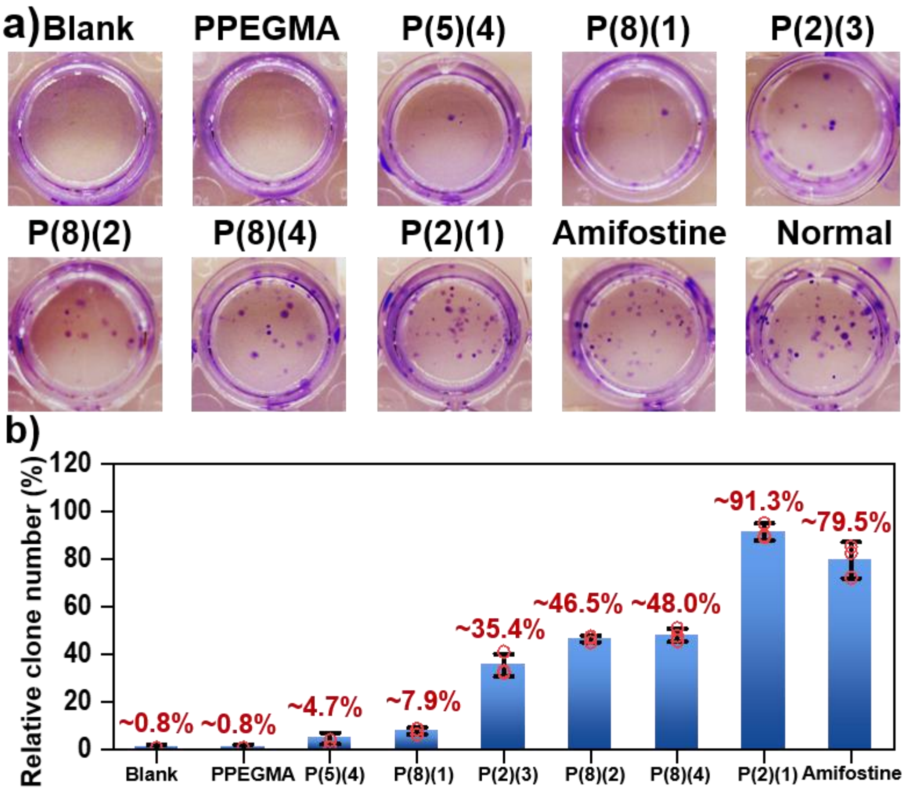

Figure S10: Colony formation assay of L929 cells. (a) The images of cell colony under different conditions, 48 h culture: medium only (blank), P(PEGMA) (10 mg/mL), P(5)(4) (0.4 mg/mL), P(8)(1) (1 mg/mL), P(2)(3) (2 mg/mL), P(8)(2) (2 mg/mL), P(8)(4) (2 mg/mL), P(2)(1) (10 mg/mL) and amifostine (0.3 mg/mL). (b) Colony formation number under different conditions, data are presented as mean values ± SD (n = 3 independent experiments). Cells in culture medium without X-ray served as the normal group (100%). Source data are provided as a Source Data file.

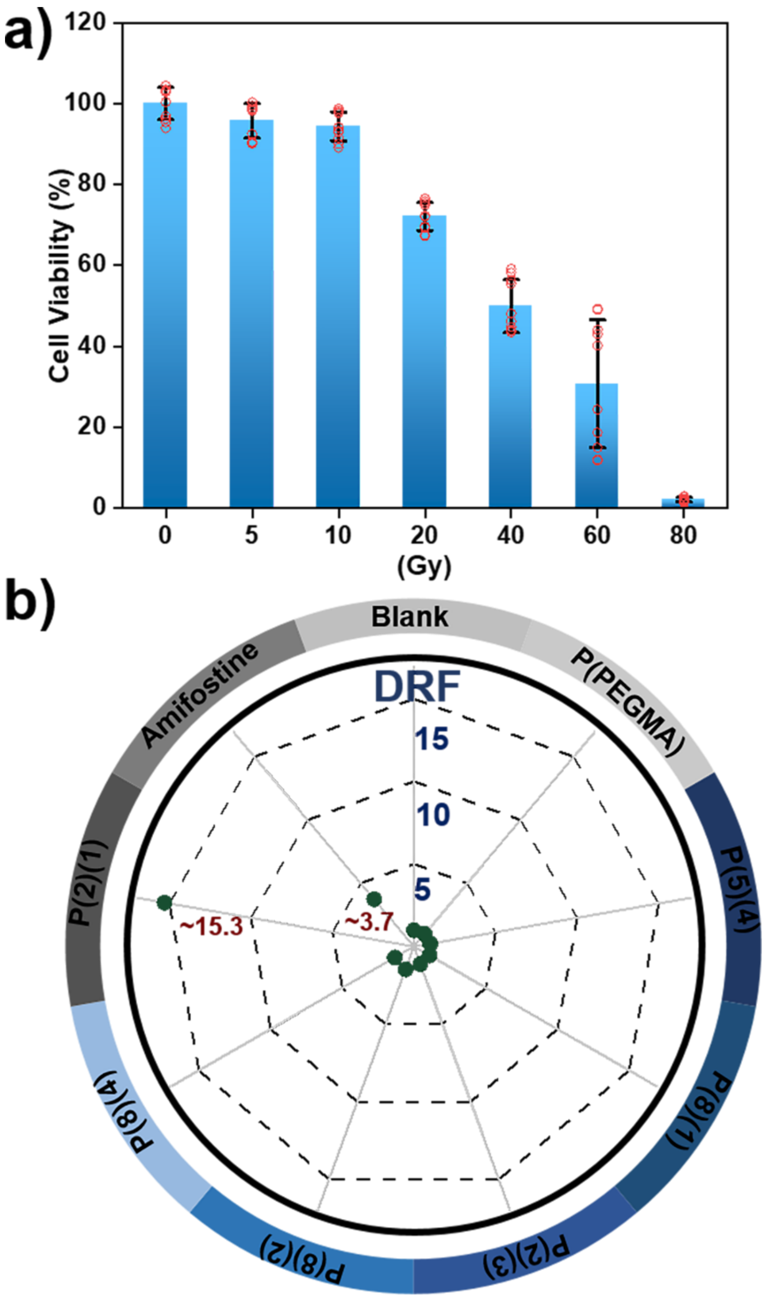

Figure S11: Cell viability and the DRF(cell) values of different compounds. (a) Viability of L929 cells after exposure to different doses of X-ray radiation, 48 h culture. Data are presented as the mean ± SD (n = 10 independent experiments). (b) The DRF(cell) values of different polymers and amifostine at their cytosafety concentrations (48 h). Source data are provided as a Source Data file.

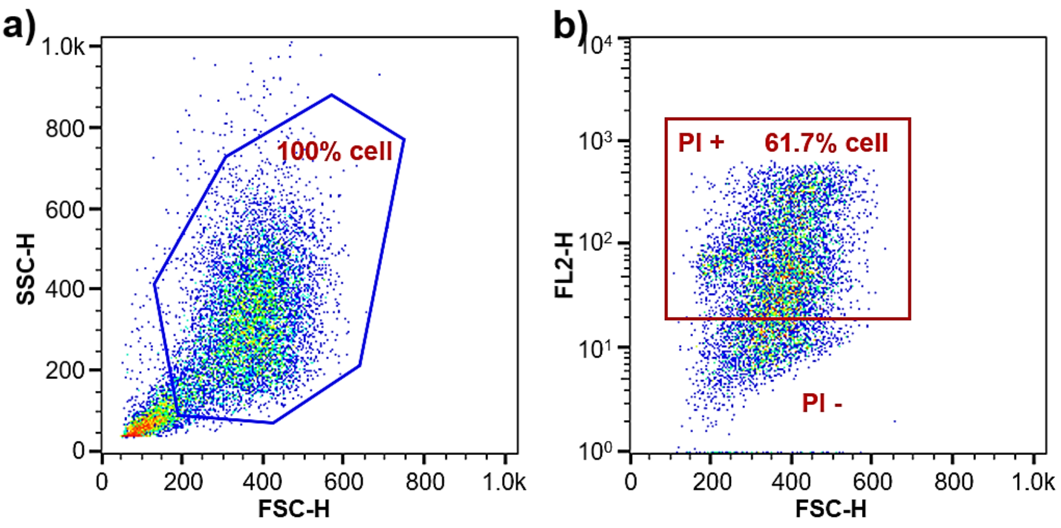

Figure S12: Figure exemplifying the gating strategy. (a) Gate (blue box) was designed based on FSC-H vs SSC-H graphs to remove debris. (b) Cell subsets expressing the PI (FL2: λex = 488 nm) were selected with gates (red box).

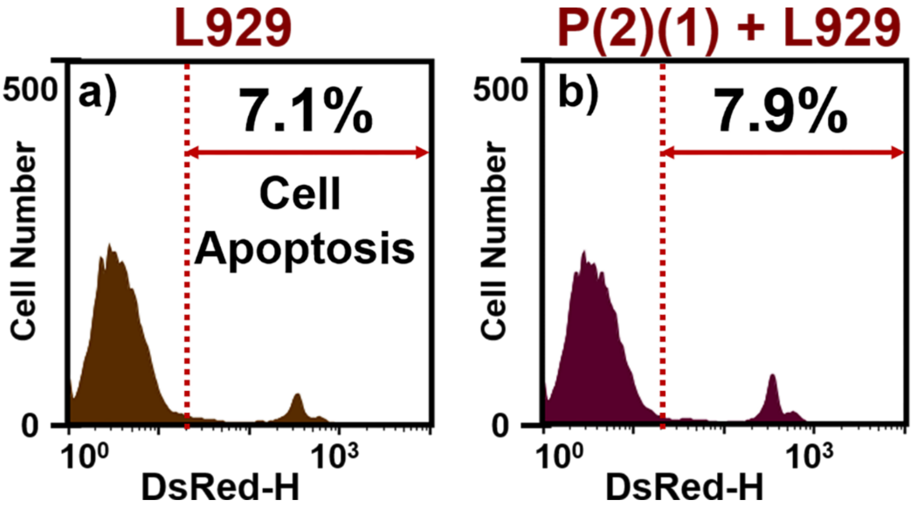

Figure S13: Flow cytometry analysis of cells. (a) Cells in a medium only (48 h) and (b) cells cultured with P(2)(1) (10 mg/mL, 48 h).

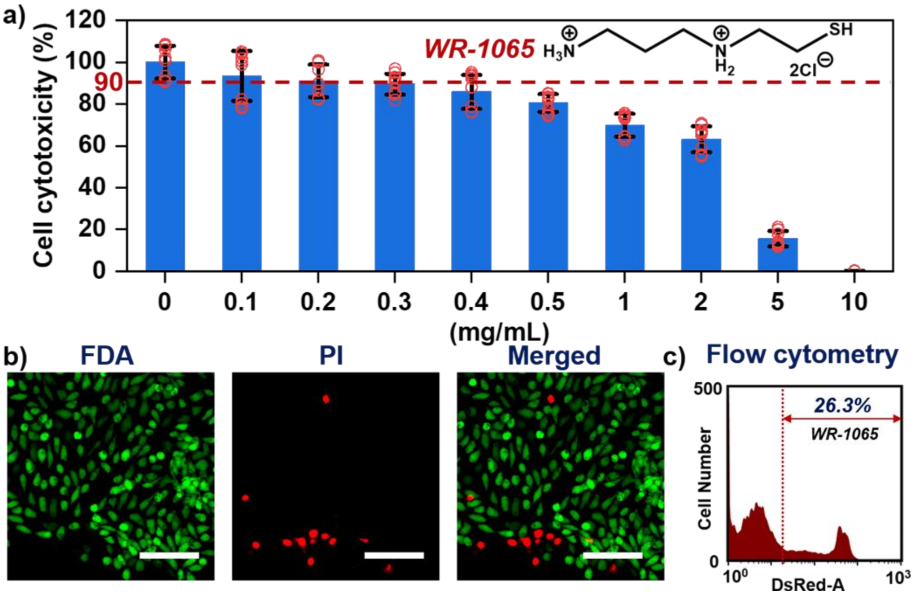

Figure S14: Cytotoxicity of WR-1065 and its radioprotection to L929 cells. (a) Cytotoxicity of WR-1065 to L929 cells, 48 h culture. Cell viability in a culture medium only was defined as 100% viability. Data are presented as mean values ± SD (n = 10 independent experiments). (b) FDA/PI double staining of L929 cells after exposure to 80 Gy X-ray radiation with WR-1065 (0.3 mg/mL). Scale bar = 100 µm. (c) Flow cytometry analysis of cell necrosis with WR-1065 (0.3 mg/mL). Source data are provided as a Source Data file.

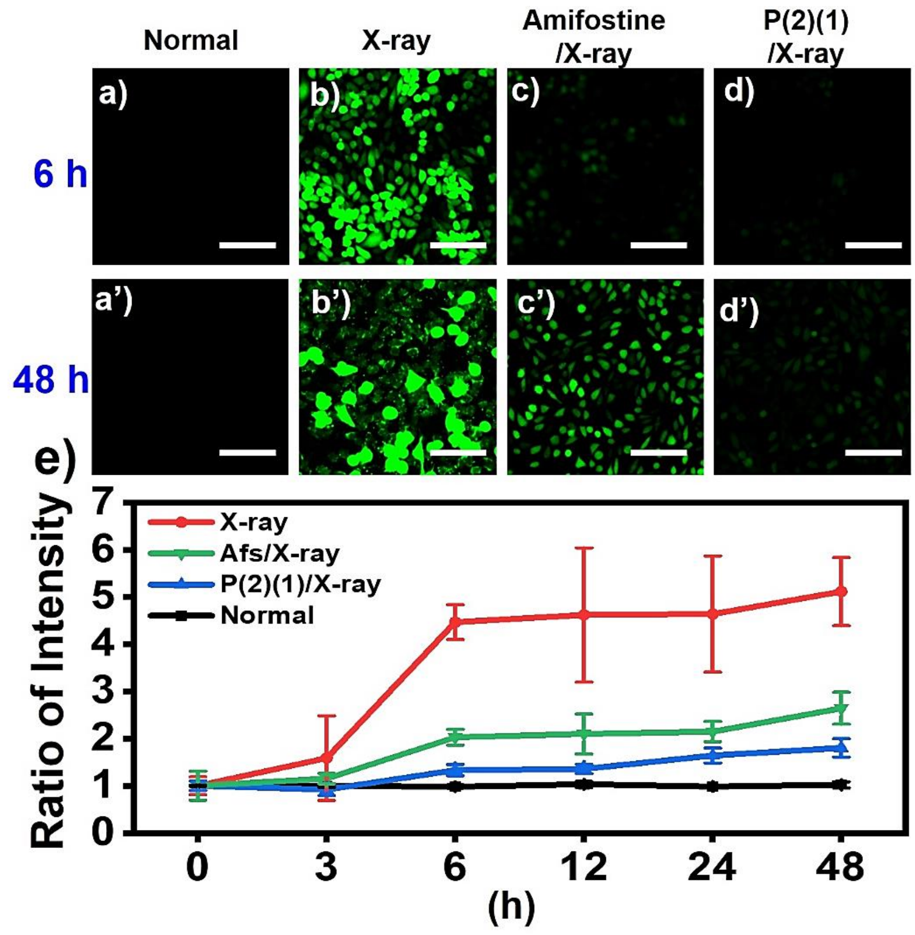

Figure S15: ROS analysis results. ROS level images (DCFH-DA as fluorescence probe) of different cells at different time. (a, a’) Normal cells (a: 6 h, a’: 48 h). (b, b’) Cells after exposure to X-ray radiation (b: 6 h, b’: 48 h). (c, c’) Cells with amifostine (0.3 mg/mL) after exposure to X-ray radiation (c: 6 h, c’: 48 h). (d, d’) Cells with P(2)(1) (10 mg/mL) after exposure to X-ray radiation (d: 6 h, d’: 48 h). Scale bar = 100 µm. (e) ROS levels (DCFH-DA as a probe) in L929 cells at different time points. Data are presented as mean values ± SD (n = 5 independent experiments). Source data are provided as a Source Data file.

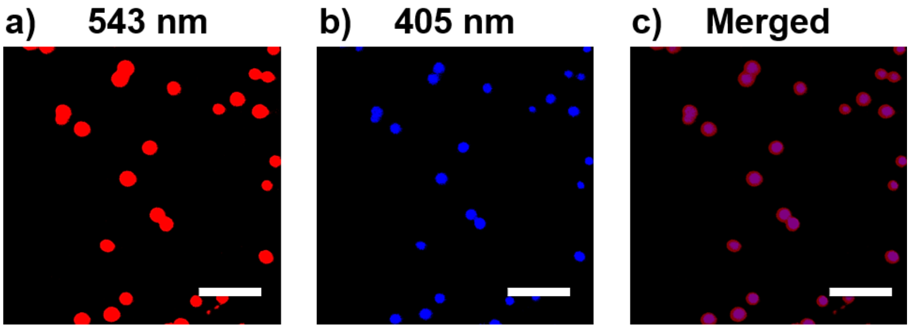

Figure S16: Images of cells after the comet assay. (a) λ_{ex} = 543 nm. (b) λ_{ex} = 405 nm; blue fluorescence stemming from the 1,4-DHP group in P(2)(1). (c) Merged image. Scale bar = 200 µm. This experiment was repeated three times independently with similar results.

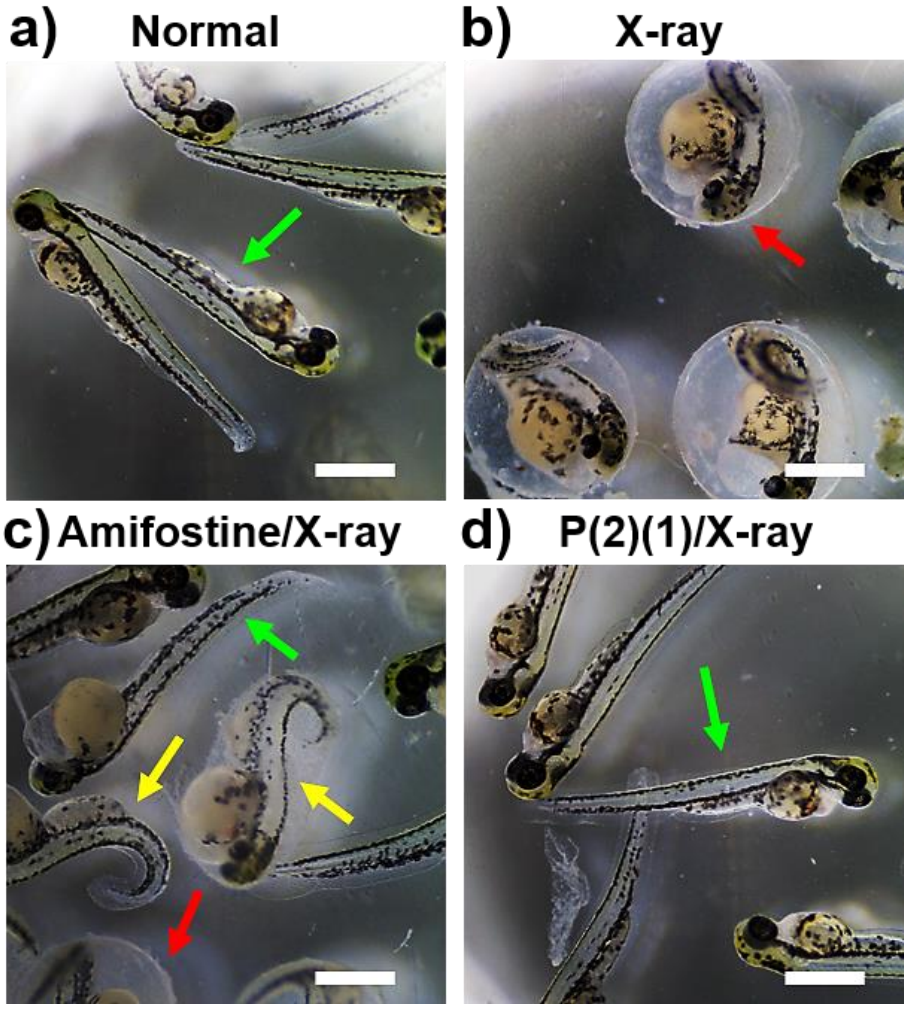

Figure S17: Representative pictures of zebrafish larvae hatched from embryos under different conditions. (a) Normal (without X ray). (b) X-ray (80 Gy). (c) X-ray (80 Gy) with amifostine (0.3 mg/mL). (d) X-ray (80 Gy) with P(2)(1) (10 mg/mL). Green arrows: larvae with straight spines (normal). Yellow arrows: larvae with curved spines (deformed). Red arrows: unhatched embryos. Scale bar = 1 mm.

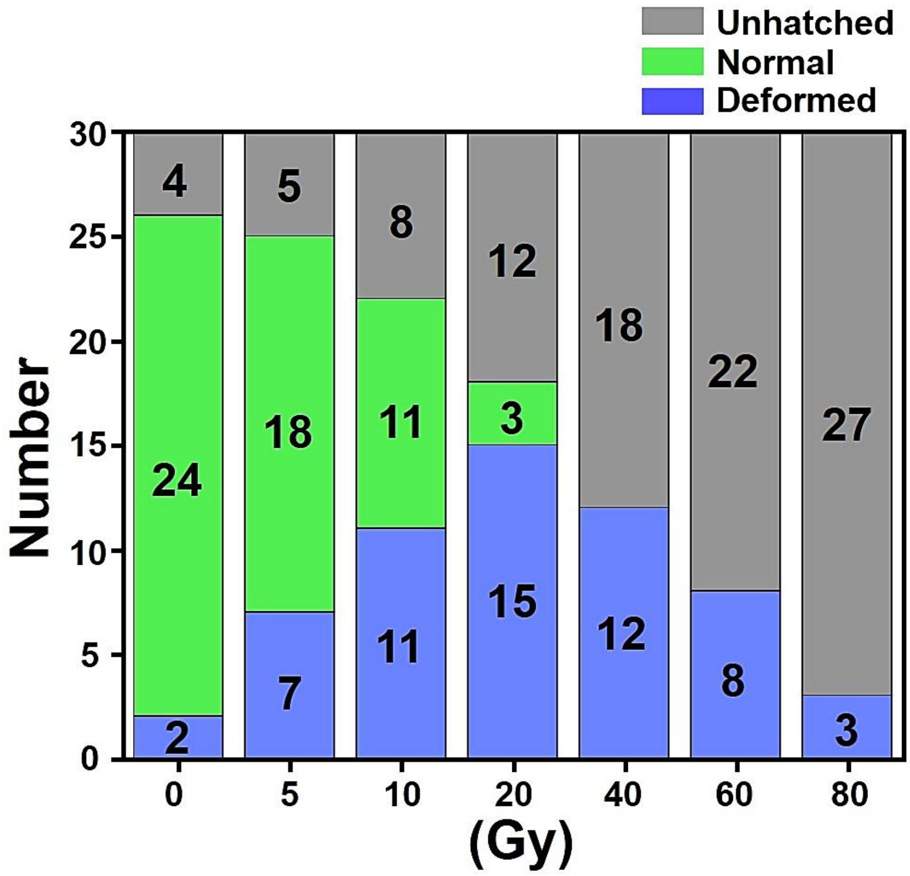

Figure S18: Numbers of different larvae. The number of unhatched embryo (gray), normal larvae (straight spine, green) and deformed larvae (curved spine, blue) after exposure to different doses of X-ray radiation, 30 samples/group. Source data are provided as a Source Data file.

Table S1: Hantzsch-type copolymers (P(X)(Y)).

Polymer	Conversion[a]	1,4-DHP/PEGMA[a]	M_n[b]	PDI[b]
P(1)(1)	98%	0.95	55000	2.19
P(2)(1)	95%	0.98	74300	3.34
P(3)(1)	99%	1.05	83300	3.18
P(4)(1)	99%	1.06	110900	9.04
P(5)(1)	99%	0.98	143900	6.61
P(6)(1)	99%	0.99	62000	5.08
P(7)(1)	98%	1.04	81200	3.24
P(8)(1)	96%	0.99	48800	1.31
P(9)(1)	99%	0.98	53700	2.05
P(1)(2)	98%	0.99	59700	2.33
P(2)(2)	96%	0.96	69700	2.70
P(3)(2)	97%	1.04	60000	2.15
P(4)(2)	98%	1.04	149400	9.23
P(5)(2)	99%	0.98	121400	4.48
P(6)(2)	98%	1.03	64400	4.35
P(7)(2)	99%	1.04	69300	2.28
P(8)(2)	99%	0.99	66400	1.80
P(9)(2)	99%	1.00	58400	1.54
P(1)(3)	97%	0.98	52500	2.92
P(2)(3)	96%	0.97	76000	3.27
P(3)(3)	99%	0.95	181900	5.22
P(4)(3)	98%	1.04	154000	7.11
P(5)(3)	96%	1.05	116900	4.09
P(6)(3)	99%	0.94	62200	5.07
P(7)(3)	97%	1.02	64900	2.66
P(8)(3)	93%	0.97	39700	2.74
P(9)(3)	98%	0.98	102700	2.53
P(1)(4)	98%	0.99	52100	1.80
P(2)(4)	96%	1.02	82000	3.32
P(3)(4)	98%	1.05	178900	6.94
P(4)(4)	97%	1.00	123500	3.56
P(5)(4)	99%	0.96	70000	2.02
P(6)(4)	99%	1.02	80900	4.18
P(7)(4)	97%	1.04	56500	1.80
P(8)(4)	95%	0.99	62300	1.85
P(9)(4)	99%	0.98	102600	2.60
P(1)(5)	95%	1.05	54800	1.83
P(2)(5)	97%	1.05	68300	2.82
P(3)(5)	99%	0.98	171100	5.85
P(4)(5)	97%	1.03	186000	6.80
P(5)(5)	99%	1.02	38600	1.60
P(6)(5)	96%	1.05	72300	3.78
P(7)(5)	98%	0.98	49100	1.68
P(8)(5)	96%	0.99	66100	1.94
P(9)(5)	96%	0.96	93600	2.30

a. Calculated by ^{1}H NMR (DMSO-d_6, 400 MHz).

b. Measured by GPC using DMF as an eluent (1 mL/min).

Table S2: Anti-HOR, anti-SOR and anti-GOR ability of polymers[a].

Polymer	HOR (%)	SOR (%)	GOR (%)
P(1)(1)	36.4	43.3	26.8
P(2)(1)	87.1	96.7	99.9
P(3)(1)	19.6	52.7	0.01
P(4)(1)	54.9	69.4	50.0
P(5)(1)	68.7	82.8	42.9
P(6)(1)	11.7	49.0	32.1
P(7)(1)	34.0	43.2	44.6
P(8)(1)	91.5	97.6	100.0
P(9)(1)	38.3	71.3	42.9
P(1)(2)	63.0	76.3	50.0
P(2)(2)	68.2	89.7	50.0
P(3)(2)	65.4	76.1	60.7
P(4)(2)	73.4	74.3	99.9
P(5)(2)	50.8	83.8	78.6
P(6)(2)	53.1	78.6	19.6
P(7)(2)	32.7	80.4	33.9
P(8)(2)	93.1	98.0	100.0
P(9)(2)	75.2	87.7	76.8
P(1)(3)	34.7	54.8	60.7
P(2)(3)	89.0	96.0	87.4
P(3)(3)	37.2	47.1	67.9
P(4)(3)	74.5	68.6	76.8
P(5)(3)	23.1	61.4	66.1
P(6)(3)	28.5	61.5	3.6
P(7)(3)	30.9	53.0	41.1
P(8)(3)	90.3	83.6	100.0
P(9)(3)	32.2	70.2	64.3
P(1)(4)	29.0	46.6	37.5
P(2)(4)	42.5	74.6	55.4
P(3)(4)	28.4	46.4	50.0
P(4)(4)	76.3	62.5	80.4
P(5)(4)	88.1	95.0	96.4
P(6)(4)	44.1	85.1	67.9
P(7)(4)	30.6	57.2	80.4
P(8)(4)	91.0	90.7	100.0
P(9)(4)	45.0	65.3	55.4
P(1)(5)	25.6	42.5	64.3
P(2)(5)	31.5	73.5	44.6
P(3)(5)	19.4	43.0	30.4
P(4)(5)	81.8	91.8	87.5
P(5)(5)	24.8	52.7	48.2
P(6)(5)	29.8	70.0	14.3
P(7)(5)	31.5	48.3	44.6
P(8)(5)	87.0	82.5	100.0
P(9)(5)	32.6	74.6	57.1

a. A PBS solution (100 μL, pH 7.4) was used as a blank (0%).
