# Supplementary material for: High-throughput preparation of radioprotective polymers via Hantzsch’s reaction for in vivo X-ray damage determination
Source: Nat Commun. 2020 Dec 4;11:6214. doi: 10.1038/s41467-020-20027-0 (PMC7718248; doi:10.1038/s41467-020-20027-0)
Supplement: Supplementary file 3 — Reporting Summary [file 41467_2020_20027_MOESM3_ESM.pdf]

nature research

Corresponding author(s): Lei Tao

Last updated by author(s): Oct 20, 2020

Reporting Summary

Nature Research wishes to improve the reproducibility of the work that we publish. This form provides structure for consistency and transparency in reporting. For further information on Nature Research policies, see our Editorial Policies and the Editorial Policy Checklist.

Statistics

For all statistical analyses, confirm that the following items are present in the figure legend, table legend, main text, or Methods section.

n/a	Confirmed	
☐	☒	The exact sample size (*n*) for each experimental group/condition, given as a discrete number and unit of measurement
☐	☒	A statement on whether measurements were taken from distinct samples or whether the same sample was measured repeatedly
☐	☒	The statistical test(s) used AND whether they are one- or two-sided<br>*Only common tests should be described solely by name; describe more complex techniques in the Methods section.*
☒	☐	A description of all covariates tested
☐	☒	A description of any assumptions or corrections, such as tests of normality and adjustment for multiple comparisons
☐	☒	A full description of the statistical parameters including central tendency (e.g. means) or other basic estimates (e.g. regression coefficient) AND variation (e.g. standard deviation) or associated estimates of uncertainty (e.g. confidence intervals)
☐	☒	For null hypothesis testing, the test statistic (e.g. *F*, *t*, *r*) with confidence intervals, effect sizes, degrees of freedom and *P* value noted<br>*Give P values as exact values whenever suitable.*
☒	☐	For Bayesian analysis, information on the choice of priors and Markov chain Monte Carlo settings
☒	☐	For hierarchical and complex designs, identification of the appropriate level for tests and full reporting of outcomes
☒	☐	Estimates of effect sizes (e.g. Cohen's *d*, Pearson's *r*), indicating how they were calculated

Our web collection on statistics for biologists contains articles on many of the points above.

Software and code

Policy information about availability of computer code

Data collection	LSCM: ZEN black 2.3; NMR: Delta 5.3.1; a BD Calibur flow cytometer
Data analysis	Statistical analysis：SPSS Statistics v.25.0 and MedCalc 18.1; LSCM: ImageJ 1.51; NMR: MestReNova 12.0; Flow cytometry: FlowJo V10;

For manuscripts utilizing custom algorithms or software that are central to the research but not yet described in published literature, software must be made available to editors and reviewers. We strongly encourage code deposition in a community repository (e.g. GitHub). See the Nature Research guidelines for submitting code & software for further information.

Data

Policy information about availability of data

All manuscripts must include a data availability statement. This statement should provide the following information, where applicable:

- Accession codes, unique identifiers, or web links for publicly available datasets
- A list of figures that have associated raw data
- A description of any restrictions on data availability

The authors declare that the data supporting the findings of this study are available within the paper and its supplementary information files. The source data underlying Figs 3, 5d, 6b, 6c, 7b and Supplementary Figs 6, 7, 8b, 9, 10b, 11a, 11b, 14a, 15b, 18 are provided as a Source Data file. The source data underlying Figs 3, 5d, 6b, 6c, 7b and Supplementary Figs 6, 7, 8b, 9, 10b, 11a, 11b, 14a, 15b, 18 are available in the figshare repository https://doi.org/10.6084/m9.figshare.12999419.v2.

Field-specific reporting

Please select the one below that is the best fit for your research. If you are not sure, read the appropriate sections before making your selection.

- [x] Life sciences
- [ ] Behavioural & social sciences
- [ ] Ecological, evolutionary & environmental sciences

For a reference copy of the document with all sections, see nature.com/documents/nr-reporting-summary-flat.pdf

Life sciences study design

All studies must disclose on these points even when the disclosure is negative.

Sample size	Sample sizes were chosen based on previous experience and on what is common practice in the field. Statistical analyses of results were performed by Student's t-test for independent samples (one-side), the sample sizes were suitable for the statistical analyses.
Data exclusions	No sample was optionally excluded from the analysis.
Replication	All experiments were independently repeated as indicated and were reliably reproduced (see Figure Legends where applicable for further details). Biological replicates are defined as independent experiments performed on different biological samples.
Randomization	For all L929 cells, fish embryos and zebrafish larvae, all samples were randomly selected throughout this study.
Blinding	Blinding was not performed during experiments to facilitate staff monitoring.

Reporting for specific materials, systems and methods

We require information from authors about some types of materials, experimental systems and methods used in many studies. Here, indicate whether each material, system or method listed is relevant to your study. If you are not sure if a list item applies to your research, read the appropriate section before selecting a response.

Materials & experimental systems

n/a	Involved in the study	
[ ]	[x]	Antibodies
[ ]	[x]	Eukaryotic cell lines
[x]	[ ]	Palaeontology and archaeology
[ ]	[x]	Animals and other organisms
[x]	[ ]	Human research participants
[x]	[ ]	Clinical data
[x]	[ ]	Dual use research of concern

Methods

n/a	Involved in the study	
[x]	[ ]	ChIP-seq
[ ]	[x]	Flow cytometry
[x]	[ ]	MRI-based neuroimaging

Antibodies

Antibodies used	Phospho-Histone H2AX (Ser139) Rabbit Monoclonal Antibody (Beyotime): Category: Rabbit Monoclonal Antibody (RabMAb); Primary antibody; Product ID: AF1201; Isotype: IgG.<br>Alexa Fluor 555-Labeled Donkey Anti-Rabbit IgG (Beyotime), Product ID: A0453
Validation	All commercial antibodies had validation statements and results on the manufacturer's websites.

Eukaryotic cell lines

Policy information about cell lines

Cell line source(s)	L929 cells were purchased from ATCC
Authentication	None of cells used were authenticated.
Mycoplasma contamination	The cell lines were not tested for Mycoplasma contamination
Commonly misidentified lines (See ICLAC register)	No commonly misidentified line was used in this study.

Animals and other organisms

Policy information about studies involving animals; ARRIVE guidelines recommended for reporting animal research

Laboratory animals	This study used laboratory animal (zebra fish): Tuebingen (Tu) strain, no selective gender in this study.
Wild animals	This study did not involve wild animals.
Field-collected samples	This study did not involve samples collected from field.
Ethics oversight	All the experimental procedures involving zebrafish were approved by the Animal Care and Use Committee of Tsinghua University.

Note that full information on the approval of the study protocol must also be provided in the manuscript.

Flow Cytometry

Plots

Confirm that:

- [x] The axis labels state the marker and fluorochrome used (e.g. CD4-FITC).
- [x] The axis scales are clearly visible. Include numbers along axes only for bottom left plot of group (a 'group' is an analysis of identical markers).
- [x] All plots are contour plots with outliers or pseudocolor plots.
- [x] A numerical value for number of cells or percentage (with statistics) is provided.

Methodology

Sample preparation	After exposed to X-ray and cultured with different compounds for 48 h, L929 cells were added a PBS solution of PI (10 µg/mL) and kept for 15 min. Then, cells were washed twice by PBS (pH = 7.4) followed by collection by trypsin digestion and centrifugation (1000 rpm, 5 min). The flow cytometry analyses were performed on a BD Calibur flow cytometer. The fluorescence intensities of L929 cells were recorded according to the fluorescence signal of Cascade-red channel (λex = 488 nm), total cell number is ~10^4.
Instrument	BD Calibur
Software	BD Calibur; FlowJo
Cell population abundance	Cells (10^4) were analyzed with post-sort populations. Cells were gated by FSC and SSC to discard cell adhesion and debris.
Gating strategy	The gating strategy was chose according to reported literatures. A figure exemplifying the gating strategy was added in supplementary information

- [x] Tick this box to confirm that a figure exemplifying the gating strategy is provided in the Supplementary Information.
